# Multivariable prediction models of caries increment: a systematic review and critical appraisal

**DOI:** 10.1186/s13643-023-02298-y

**Published:** 2023-10-30

**Authors:** Kristian Havsed, Gunnel Hänsel Petersson, Per-Erik Isberg, Maria Pigg, Gunnel Svensäter, the Foresight Research Consortium, Madeleine Rohlin

**Affiliations:** 1 Department of Pediatric Dentistry, Institute for Postgraduate Dental Education, Jönköping, Sweden; 2 Centre for Oral Health, School of Health and Welfare, Jönköping University, Jönköping, Sweden; 3 Faculty of Odontology, Malmö University, Malmö, Sweden; 4 Department of Statistics, Lund University, Lund, Sweden

**Keywords:** CHARMS, Dental caries, Likelihood ratio, Prediction models, PROBAST

## Abstract

**Background:**

Multivariable prediction models are used in oral health care to identify individuals with an increased likelihood of caries increment. The outcomes of the models should help to manage individualized interventions and to determine the periodicity of service. The objective was to review and critically appraise studies of multivariable prediction models of caries increment.

**Methods:**

Longitudinal studies that developed or validated prediction models of caries and expressed caries increment as a function of at least three predictors were included. PubMed, Cochrane Library, and Web of Science supplemented with reference lists of included studies were searched. Two reviewers independently extracted data using CHARMS (Critical Appraisal and Data Extraction for Systematic Reviews of Prediction Modelling Studies) and assessed risk of bias and concern regarding applicability using PROBAST (Prediction model Risk Of Bias ASessment Tool). Predictors were analysed and model performance was recalculated as estimated positive (LR +) and negative likelihood ratios (LR −) based on sensitivity and specificity presented in the studies included.

**Results:**

Among the 765 reports identified, 21 studies providing 66 prediction models fulfilled the inclusion criteria. Over 150 candidate predictors were considered, and 31 predictors remained in studies of final developmental models: caries experience, mutans streptococci in saliva, fluoride supplements, and visible dental plaque being the most common predictors. Predictive performances varied, providing LR + and LR − ranges of 0.78–10.3 and 0.0–1.1, respectively. Only four models of coronal caries and one root caries model scored LR + values of at least 5. All studies were assessed as having high risk of bias, generally due to insufficient number of outcomes in relation to candidate predictors and considerable uncertainty regarding predictor thresholds and measurements. Concern regarding applicability was low overall.

**Conclusions:**

The review calls attention to several methodological deficiencies and the significant heterogeneity observed across the studies ruled out meta-analyses. Flawed or distorted study estimates lead to uncertainty about the prediction, which limits the models’ usefulness in clinical decision-making. The modest performance of most models implies that alternative predictors should be considered, such as bacteria with acid tolerant properties.

**Trial registration:**

PROSPERO CRD#152,467 April 28, 2020

**Supplementary Information:**

The online version contains supplementary material available at 10.1186/s13643-023-02298-y.

## Background

Prediction models are used to estimate the probability of the presence of a particular disease (diagnosis) or to estimate the probability of developing a particular outcome in the future (prognosis) [1]. Estimates of probabilities of developing an outcome are rarely based on a single predictor and care providers naturally integrate several variables [1].

Dental caries, defined as bacteria-triggered localised demineralization of dental tissues, is estimated to have a global prevalence of 35% and is associated with high societal costs [2]. A prediction model of caries involves an assessment of the probability that a number of new lesions will occur over time. The model output will help to realize individualized preventive interventions and to determine the periodicity of service. Since many clinicians apply prediction models of caries daily, critical appraisal of models is crucial. Recent evidence suggests that there is a need to improve the methodological standards, and predictive analytic methods with alternative predictors are called for [3]. Still, it is important to update facts about predictors presented in current scientific literature, and not to squander information from previous studies.

The purpose of systematic reviews (SRs) is to compile, analyse and interpret all available data to make reliable conclusions, and to identify knowledge gaps. The CHecklist for critical Appraisal and data extraction for systematic Reviews of prediction Modelling Studies (CHARMS) was designed to guide the framing of review questions of SRs and the extraction of relevant items of prediction model studies [4]. For the assessment of risk of bias and applicability, which are essential steps in any SR, the Prediction model Risk Of Bias ASsessment Tool (PROBAST) was developed [5, 6]. The objective of this study was to systematically review and critically appraise studies of development and validation of multivariable predictive models for assessment of caries increment *i.e.,* caries onset or caries progression with the aid of CHARMS and PROBAST. In particular, we aimed to focus on the predictors, risk of bias, and the predictive performance.

## Methods

### Design

We followed the PRISMA 2020 checklist [7] (Additional file 1). Prior to the formal start of the study, the review protocol was registered with the University of York Centre for Reviews and Dissemination International prospective register of systematic reviews (PROSPERO) (submitted October 3, 2019; registration April 28, 2020, Registration #152,467), and later supplemented (November 30, 2020) with a checklist based on CHARMS.

### Eligibility criteria

*Inclusion criteria* were based on PICOTS (Participants, Intervention, Comparator, Outcome, Timing, Setting). Studies were included if they met the following criteria:

- Study design: longitudinal prospective or retrospective study.
- Participants: individuals of all ages, sex, and ethnicity. Caries should be defined at baseline and follow-up regarding prevalence and severity on an individual basis. Alternatively, caries progression should be possible to calculate from data presented in the included study or in studies referred to.
- Intervention: a prediction model that expresses caries increment as a function of at least 3 variables as predictors. Predictors described in sufficient detail to allow calculation of model performance. When predictors were not described in detail but referred to, the referenced study was retrieved to recover key data.
- Comparator: additional prediction model(s) included in the study.

Outcome to be predicted: development either (i) from sound tooth/tooth surface to detectable lesion in enamel or dentin: i.e., from health to disease onset, or (ii) from initial to more extensive lesion: i.e., individual caries progression, described with thresholds to allow calculation of model performance. When not described but referred to, the referenced study was retrieved to recover key data. The outcome may be phrased as caries, caries experience, caries increment, or caries progression. In the following text, the term *caries increment* is defined as the number of new lesions, teeth or surfaces occurring in an individual within a stated period of time [8].

- Timing: follow-up time ≥ 1 year.
- Setting: oral health care without restriction to geographical location.
- Model performance: calibration, discrimination (e.g., AUC, area under receiver operating curve, equivalent to *c-*statistics) and classification measures (e.g., sensitivity, specificity, positive and negative predictive values, positive (LR +) and negative (LR −) likelihood ratios [4]. Measure values should be correctly calculated and presented based on data described in the study and with data allowing recalculation of model performance with confidence interval. *C-*statistics assessing discrimination was not accepted as the only performance measure [4].
- Language: English.

*Exclusion criteria* were as follows:

- multivariable prediction model(s) of caries increment were not presented
- was not original research (e.g., non-systematic reviews, letters, editorials)
- included ≤ 2 variables in final prediction model(s)
- model performance or only AUC were not presented
- narrative reviews, case report or case series.

### Information sources and search strategy

Three databases were searched (MEDLINE via PubMed, Web of Science, and the Cochrane Library in Cochrane Database of Systematic Reviews) from 1966 up to April 23, 2021. Reference lists of included publications and 4 systematic reviews of prediction models of caries increment [9–12] were screened to identify additional studies of potential interest. We also searched the PROSPERO database on October 3, 2019, to identify any upcoming reviews.

The search plan was managed with the aid of university librarians. The MEDLINE search is presented in Additional file 2. The Web of Science search was performed in all citation databases. PubMed and Web of Science searches were screened for duplicate publications by manual search.

### Selection process

The selection of studies was completed in 2 phases. In phase one, all retrieved records were independently assessed according to title and/or abstract by 2 review authors and selected according to the eligibility criteria. Records selected by at least one reviewer were retrieved in full text for further selection. In phase 2, two review authors independently included or excluded full text publications using a piloted protocol. The protocols were compared and discussed. Disagreements were resolved by involving a third review author.

### Data collection process

A data extraction form based on CHARMS, tailored according to the review objective was developed. The form was piloted using five publications among four review authors, who filled out the form independently. The results of the extraction were discussed between the review authors and the extraction form was adjusted after discussion. Subsequently, two teams of two review authors independently extracted key characteristics of the included studies using the extraction form.

### Data items

Information on each study, as presented in Table 1 and Additional file 4 was collected [13–33]. The event count per candidate predictor was calculated from the study information. Results of data extraction of each publication were discussed among two reviewers and disagreements were resolved by involving a third review author, and a common protocol for the reviewers was established. Thence, information of predictors and predictive model performances in particular were reviewed once more by four review authors. In case of inconsistencies, attempts were made to contact the corresponding authors for clarification. When no reply was received, the data were presented narratively or not at all. Regarding the model development, the number of candidate predictors and methods used to select predictors in final models were collected. For each model, predictors included in the final model and model performance were extracted (Table 2) [13–35].

**Table 1:** Main characteristics of included studies of multivariable prediction models of caries increment. A detailed description of included studies is presented in Additional file 4

First author and year [ref]	Country Year	Age (years) at baseline	Outcome (caries increment) Method for measurement	Sample size	Number of events (E)	Events per variable (EPV)
**Studies of model development**						
* Coronal caries*						
Angulo 1995 [13]	Uruguay 1988–1990	12–13	DS ˃ 1 cavity Visual-tactile examination	69	19	6.33
Demers 1992 [14]	Canada 1988	Mean 5	dmfs ˃ 0 dentine Visual-tactile examination	302	143	15.9
Disney 1992 [15]	USA 1986–1989	6 and 10	- DMFS ≥ 2 dentine - DMFS ≥ 4 dentine Visual-tactile examination	965–1099	204–234	5.2–5.6
Fontana 2011 [16]	Puerto Rico 2007	5–13	- ICDAS ≥ 1enamel - ICDAS ≥ 3 cavity Visual-tactile examination and bitewing radiography	395	239–35	5.8–8.7
Gao 2010 [17]	Singapore 2009–2010	3–6	dmft ˃ 0 dentine Visual-tactile examination	1576	689	57–114.8
Hänsel Petersson 2002 [18]	Sweden 1998	10–11	DMFS/DMFT > 0 dentine Dental records with bitewing radiography	392	121	10
Pang 2021 [19]	China 2018–2020	13–14	ICDAS ≥ 3 cavity Visual-tactile examination	633	365	7.7
Sánchez-Pérez 2009 [20]	Mexico 2001–2007	6	dmfs/DMFS ≥ 1 dentine Visual-tactile examination	95	56	5.1
* Coronal and root caries*						
Powell 1991 [21]	USA NR	66–95	≥ 1 coronal and/or root lesion Visual-tactile examination	21	16	0.7
* Root caries*						
Ritter 2016 [22]	USA 2007–2008	Mean 52	Any incident root caries Visual-tactile examination	155	76	5.8–9.5
Sánchez-García 2011 [23]	Mexico 2004–2005	Mean 73	≥ 1 root surfaces Visual-tactile examination	531	115	3.7
**Studies of model validation**						
* Coronal caries*						
Beck 1992 [24]	USA 1986–1989	6, 10	- DMFS ≥ 2 dentine - DMFS ≥ 4 dentine Visual-tactile examination	965–1099	338–642	8.7–14.6
Birpou 2019 [25]	Greece NR	2–5	“Sound to non-cavitated” + “non-cavitated to cavitated” Visual-tactile examination	140–147	74–77	8.2–10.1
Campus 2012 [26]	Italy 2007–2009	7–9	DFS ˃ 0 cavity Visual-tactile examination	861	469	67
Christian 2020 [27]	Australia NR	1.5	ICDAS-II ˃ 0 cavity Visual examination	214	39–75	3.0–5.8
Dolic 2020 [28]	Bosnia and Herzegovina 2007–2011	Mean 27	DMFT ˃ 1 cavity Visual-tactile examination	80	5	6
Gao 2013 [29]	Hong Kong NR	3	dmft ˃ 0 cavity Visual-tactile examination	485	178	16.2–35.6
Hänsel Petersson 2015 [30]	Sweden 2006–2007	19	DFS ≥ 1 dentine Visual-tactile examination, bitewing radiography	982	344	4.1–13.66
Hänsel Petersson 2010 [31]	Sweden 1998–2000	10–11	DMFS ˃ 0 dentine Dental records and bitewing radiography	392	122	13.5–20.3
Lif Holgerson 2009 [32]	Sweden 2002–2007	2	dmfs/DMFS > 0 enamel and dentine Visual-tactile examination and bitewing radiography	55	20	2.9
Pang 2020 [19]	China 2018–2020	13–14	ICDAS ≥ 3 cavity Visual-tactile examination	320	202	4.4
* Root caries*						
Hayes 2018 [33]	Ireland 2012–2015	≥ 65	≥ 1 root surface with cavity Visual-tactile examination	280	70	7.8–11.7

*Abbreviations*: *DMFT* decayed missing filled teeth (permanent), *DMFS* decayed missing filled surfaces (permanent), *DS* decayed surfaces (permanent), *dmft* decayed missing filled teeth (primary), *dmfs* decayed missing filled surfaces (primary), *NR* not reported

**Table 2:** Included predictors and model performance of final multivariable prediction models of caries increment

First author, Year of publication [reference]	Predictors in final model Number (*N*) Predictor levels: • *Societal structural* • *Life-style situational* • *Physiological* • *Oral biological* • *Tooth* • *Caries experience* • *Other predictors*	Model performance (according to included study) Classification measures: - Sensitivity (Sens) - Specificity (Spec) - Positive predictive value (PPV) - Negative predictive value (NPV) - Positive likelihood ratio (LR +) - Negative likelihood ratio (LR −) Discrimination: - Area under curve (AUC)	Positive likelihood ratio (LR +) Negative likelihood ratio (LR −) calculated by review authors based on sensitivity and specificity presented in included study (95% confidence intervals)	Model interpretation by study authors	Comments
**Studies of model development**					
*Coronal caries*					
Angulo, 1995 [13]	*N* = 3 *• Oral biological* (˃ 10^4^ ms/ml saliva, ˃ 10^4^ lbc/ml saliva) *• Caries experience* (DS ˃ 3 cavity)	Sens 0.19, Spec 0.86, PPV 0.36, NPV 0.71	LR + 1.36 (0.43–4.31) LR − 0.94 (0.74–1.20)	High ms counts + high lbc counts + Caries experience presented higher specificity but lower sensitivity than Caries experience alone	Sample with low socioeconomic status. Caries prevalence at baseline NR Caries increment (1.5 years) 45%
Demers, 1992 [14]	Model 1 (full model) *N* = 5 *• Societal structural* (parents’ education) *• Oral biological* (≥ 10^5^ ms/ml saliva, ≥ 10^5^ lbc/ml saliva debris index-screening level ≥ 1.9) *• Caries experience* (dmfs ˃ 0 cavity) Model 2 (Socioeconomic model) *N* = 3 *• Societal structural* (parents’ education, family structure) *• Life-style situational* (fluoride supplements)	Model 1 (full model) Sens 0.94, Spec 0.41 Model 2 (socioeconomic model) Sens 0.86, Spec 0.35	Model 1 (full model) LR + 1.60 (1.40–1.83) LR − 0.14 (0.07–0.28) Model 2 (socioeconomic model) LR + 1.31 (1.15–1.50) LR − 0.40 (0.26–0.64)	Caries experience reached sensitivity and specificity values close to the best model over 1 year. More predictors add to costs and complexity of screening	Caries prevalence at baseline NR Caries increment (1 years) 47%
Disney, 1992 [15]	Aiken *N* = 23; Portland *N* = 22 *• Societal structural* (referral caries score) *• Life-style situational* (between-meal snacks, Tooth-brushing–2 predictors, F-rinse) *• Physiological* (age, race) *• Oral biological* (ms in saliva, lbc in saliva, plaque score) *• Tooth* (tooth morphology, fluorosis, sound permanent surfaces, sealants)*• Caries experience* (dmfs, DMFS, white spot lesions) *• Other predictors* (examiner agreement–4 predictors, predicted caries score, fluorosis x white spot lesions)	Aiken age 6.6 years Sens 0.59, Spec 0.83 Portland age 6.9 years Sens 0.59, Spec 0.84 Aiken age 10.7 years Sens 0.62, Spec 0.81 Portland age 10.8 years Sens 0.62, Spec 0.84	Aiken age 6.6 years LR + 3.47 (2.89–4.16) LR − 0.49 (0.42–0.58) Portland age 6.9 years LR + 3.69 (3.05–4.45) LR − 0.49 (0.42–0.57) Aiken age 10.7 years LR + 3.26 (2.73–3.91) LR − 0.47 (0.40–0.55) Portland age 10.8 years LR + 3.88 (3.19–4.71) LR − 0.45 (0.38–0.54)	Models will be useful for predicting those at low and high risk of caries increment. Clinical predictors were the most important group ms, lbc, sociodemographic, and dental behavior data contributed little over a 3-year follow-up	Caries prevalence at baseline NR Caries increment (3 years): Aiken 6.6 years 58% 10.7 years 66% Portland 6.9 years 31% 10.8 years 46% Thresholds for ms, lbc, and some other predictors NR
Fontana, 2011 [16]	*N* = 3–7 in 11 models Predictor common for all models except for model 11 *• Societal structural* (caregiver (CG) does not consider child’s oral health to be very good) 1 year follow-up ICDAS ≥ 3 Models 1, 2, 3 Model 1 *N* = 4 *• Life-style situational* (time elapsed since last dental visit) *• Caries experience* (tooth extracted, tooth restored) Model 2 *N* = 5 Model 1 + dmfs/DMFS ICDAS ≥ 3 at end Model 3 *N* = 7 Model 1 + dmfs/DMFS ≥ 3 at start and *• Societal structural*: (CG received a referral note for child) *• Life-style situational*** (**soda drinks, soda between meals) ICDAS ≥ 1 Models 4, 5 Model 4 *N* = 3 dmfs/DMFS ICDAS ≥ 3 at end + *• Caries experience* (tooth extracted) Model 5 *N* = 3 dmfs/DMFS ICDAS ≥ 3 at start + *• Caries experience* (tooth extracted) 2 years follow-up ICDAS ≥ 3 Models 6, 7, 8 Model 6 *N* = 3 *• Life-style situational* (time elapsed since last dental visit) *• Caries experience* (tooth restored) Model 7 *N* = 4 Model 6 + dmfs/DMFS ICDAS ≥ 3 at end Model 8 *N* = 3 dmfs/DMFS ICDAS ≥ 3 at start + *• Caries experience* (child had tooth restored) ICDAS ≥ 1 Models 9, 10, 11 Model 9 *N* = 4 *• Societal structural* (CG has current caries, CG received a referral note for child) *• Caries experience* (current caries, tooth restored) Model 10 *N* = 5 Model 9 + dmfs/DMFS ICDAS ≥ 3 at end Model 11 *N* = 3 dmfs/DMFS ICDAS ≥ 3 at start + *• Societal structural* (CG has current caries, CG received a referral note for child)	1 year follow-up ICDAS ≥ 3 Model 1 Sens 0.80, Spec 0.58, AUC 0.75 Model 2 Sens 0.81, Spec 0.58, AUC 0.77 Model 3 Sens 0.81, Spec 0.57, AUC 0.79 ICDAS ≥ 1 Model 4 Sens 0.79, Spec 0.58, AUC 0.77 Model 5 Sens 0.79, Spec 0.58, AUC 0.77 2 years follow-up ICDAS ≥ 3 Model 6 Sens 0.73, Spec 0.61, AUC 0.70 Model 7 Sens 0.73, Spec 0.61, AUC 0.73 Model 8 Sens 0.73, Spec 0.61, AUC 0.70 ICDAS ≥ 1 Model 9 Sens 0.82, Spec 0.59, AUC 0.75 Model 10 Sens 0.84, Spec 0.59, AUC 0.76 Model 11 Sens 0.75, Spec 0.61, AUC 0.77	1 year follow-up ICDAS ≥ 3 Model 1 LR + 1.90 (1.57–0.31) LR − 0.34 (0.26–0.46) Model 2 LR + 1.93 (1.59–2.34) LR − 0.33 (0.24–0.44) Model 3 LR + 1.88 (1.65–0.28) LR − 0.33 (0.25–0.45) ICDAS ≥ 1 Model 4 LR + 1.88 (1.34–2.64) LR − 0.36 (0.26–0.50) Model 5 LR + 1.88 (1.34–2.64) LR − 0.36 (0.26–0.50) 2 years follow-up ICDAS ≥ 3 Model 6 LR + 1.87 (1.49–2.35) LR − 0.44 (0.35–0.56) Model 7 LR + 1.87 (1.49–2.35) LR − 0.44 (0.35–0.56) Model 8 LR + 1.87 (1.49–2.35) LR − 0.44 (0.35–0.56) ICDAS ≥ 1 Model 9 LR + 2.00 (1.35–2.95) LR − 0.31 (0.22–0.43) Model 10 LR + 2.05 (1.39–3.02) LR − 0.27 (0.19–0.39) Model 11 LR + 1.92 (1.28–2.89) LR − 0.41 (0.30–0.56)	Items related to caries experience in child or caregiver and caregiver’s rating of child’s oral health could be used to screen at-risk children (aged 5–13) in this rural population. The models were fair in their ability to predict caries	Caries prevalence at baseline NR Caries increment: -1 year 89% had ≥ 1 Surface with any Progression and 61% With progression Towards cavitation -2 years 91% had ≥ 1 Surface with any Progression and 68% With progression Towards cavitation ICDAS Ismail et al,. [34] Code 1: when seen wet no evidence of any change in colour attributable to carious activity, but after prolonged air drying a carious opacity (white or brown lesion) is visible that is not consistent with the clinical appearance of sound enamel Code 3: localized enamel breakdown because of caries with no visible dentin or underlying shadow
Gao. 2010 [17]	*N* = 6–12 predictors in 5 models Predictor common for all models • *• Physiological* (age) Prediction models Model 1 (screening) *N* = 8 *• Societal structural* (father’s education, *• Life-style situational* (between-meal-sweets) *• Physiological* (race, months of breastfeeding, no health problems) *• Oral biological* (plaque index) *• Caries experience* (dmft ˃ 2 cavities) Model 2 (full-blown) *N* = 12 *• Societal structural* (father’s education, no annual dental checks, age regarded by parents as appropriate for dental check *• Life-style situational* (months of breastfeeding, using fluorides) *• Physiological* (age, no health problems) *• Oral biological* (plaque index, ms in saliva, lbc in saliva, average pH) *• Caries experience* (dmft ˃ 2 cavities) Risk models Model 3 (screening) *N* = 7 *• Societal structural* (never lived in non-fluoridated community *• Life-style situational* (bedtime feeding, between-meal-sweets, bedtime sweets, months of breastfeeding) *• Physiological* (age) *• Oral biological* (plaque index) Model 4 (full-blown) *N* = 6 *• Life-style situational* (months of breastfeeding) *• Physiological* (age) *• Oral biological* (plaque index, ms in saliva, lbc in saliva, average pH) Model 5 Community-screening model *N* = 6 *• Societal structural* (parent’s belief about “Tooth worm”, parents do not know about bedtime milk, child’s dental status according to parents) *• Life-style situational* (using fluorides) *• Physiological* (race)	Prediction models Model 1 (screening) Sens 0.82, Spec 0.73, AUC 0.85 Model 2 (full-blown) Sens 0.90, Spec 0.90, AUC 0.96 Risk models Model 3 (screening) Sens 0.81, Spec 0.62, AUC 0.77 Model 4 (full-blown) Sens 0.83, Spec 0.92, AUC 0.95 Model 5 Community-screening model Sens 0.82, Spec 0.81, AUC 0.89	Prediction models Model 1 (screening) LR + 3.06 (2.73–3.43) LR − 0.24 (0.21–0.29) Model 2 (full-blown) LR + 9.04 (7.41–11.03) LR − 0.11 (0.08–0.13) Risk models Model 3 (screening) LR + 2.15 (1.96–2.35) LR − 0.30 (0.26–0.36) Model 4 (full-blown) LR + 10.26 (8.20–12.84) LR − 0.18 (0.16–0.22) Model 5 Community-screening model LR + 4.37 (3.80–5.04) LR − 0.22 (0.19–0.22)	Risk models were established for a range of uses in community and clinical setting	Caries prevalence at baseline 40% Caries increment (1 year) 44% (dmft)
Hänsel Petersson, 2002 [18]	*N* = 12 Cariogram *• Societal structural* (school, dental clinic) *• Life-style situational* (diet content, diet frequency, fluoride program) *• Physiological* (sex, related diseases) *• oral biological* (ms in saliva, saliva secretion, saliva buffer, plaque amount) *• Caries experience* (DMFT with cavities)	Sens 0.41, Spec 0.80	LR + 2.03 (1.48–2.79) LR − 0.74 (0.63–0.87)	Cariogram predicted caries increment in this population (aged 10–11) more accurately than any included single-factor model	Data collected as lbc counts used as a measure of cariogenic diet Predictors in oral biological and caries experience levels given a score according to a predetermined scale with 3–4 scores Caries prevalence at baseline 40% Caries increment (2 years) 31%
Pang, 2021 [19]	*N* = 7 *• Societal structural* (one-child family) *• Physiological* (sex) *• Oral biological* (plaque index, cariostat score) *• Tooth (*genetic markers rs3790506-enamel formation gene, rs1996315-water channel protein gene AQP5 *• Caries experience* (DMFT ICDAS codes 3–6 = > 0 decayed teeth)	Total AUC 0.70 (0.66–0.74) Low caries risk (DMFT ≤ 1 caries lesion) Sens 0.41, Spec 0.74, PPV 0.42, NPV 0.73 Moderate caries risk Sens 0.46, Spec 0.69, PPV 0.49, NPV 0.66 High caries risk Sens 0.54, Spec 0.69, PPV 0.74, NPV 0.48 Very high caries risk Sens 0.68, Spec 0.75, PPV 0.95, NPV 0.25	Low caries risk LR + 1.57 (1.24–1.99) LR − 0.80 (0.71–0.89) Moderate caries risk LR + 1.48 (1.20–1.82) LR − 0.79 (0.69–0.89) High caries risk LR + 1.73 (1.42–2.12) LR − 0.67 (0.58–0.77) Very high caries risk LR + 2.71 (2.18–3.38) LR − 0.43 (0.3–0.51)	Model based on both environmental and genetic factors using an algorithm based on machine learning	Caries prevalence at baseline 34% Caries increment (1.7 years) 58% Presents calibration. Study of model validation described below ICDAS Pitts and Ekstrand, [35] Code 3 = clinically detectable “cavities” limited to enamel, Codes 4,5 = clinically detectable lesions in dentine, Code 6 = lesions into pulp
Sánchez-Pérez, 2009 [20]	*N* = 4 *• Oral biological* (acid production by bacteria in saliva) *• Tooth* (total teeth present, fissure morphology) *• Caries experience* (dmfs + DMFS ≥ 1 cavity)	Sens 0.79, Spec 0.80, AUC 0.88	LR + 3.85 (2.04–7.27) LR − 0.27 (0.16–0.46)	In a developing country, caries experience was most powerful to predict caries in 6-year-old children over 3 years, but prediction was improved by fissure morphology and bacterial acid production	Caries prevalence at baseline 58% Caries increment (3 years) 59%
* Coronal and root caries*					
Powell, 1991 [21]	*N* = 3 *• Physiological* (sex) *• Oral biological* (salivary secretion) *• Caries experience* (root caries index)	Threshold ≥ l new caries Sens 0.88, Spec 0.60	Threshold ≥ l new caries LR + 2.20 (0.74–6.53) LR − 0.20 (0.04–0.90)	Model was sensible and variables easy to collect	Caries prevalence at baseline 53% Caries increment coronal and/or root caries (1 year) 76%
* Root caries*					
Ritter, 2016 [22]	*N* = 8–13 predictors in 5 models Predictors common for model 1 and all other models Model 1 *N* = 8 *• Physiological* (age, sex, race) *• Caries experience* (at-risk years, at-risk root surfaces, at-risk root surfaces squared, root caries index, root caries index squared) Model 2 *N* = 9 Model 1 + *• Life-style situational* (tobacco use) Model 3 *N* = 13 Model 1** + ** *• Life-style situational* (cereals + sugar, tea/coffee + sugar, drinks and juices, juices, sweets) Model 4 *N* = 11 Model 1 + *• Life-style situational* (toothbrush use, how often toothbrush is replaced) *• Physiological* (wears removable prosthesis–appliances) Model 5 *N* = 11 Model 1 + *• Societal structure* (income, education, insurance)	Primary data analysis (sample *n* = 155) Model 1 Sens 0.74, Spec 0.61, AUC 0.83 Model 2 Sens 0.76, Spec 0.66, AUC 0.84 Model 3 Sens 0.71, Spec 0.61, AUC 0.84 Model 4 Sens 0.74, Spec 0.62, AUC 0.83 Model 5 Sens 0.75, Spec 0.63, AUC 0.83	Model 1 LR + 1.88 (1.38–2.55) LR − 0.43 (0.29–0.66) Model 2 LR + 2.23 (1.60–3.10) LR − 0.36 (0.23–0.56) Model 3 LR + 1.81 (1.33–2.47) LR − 0.48 (0.32–0.71) Model 4 LR + 1.94 (1.42–2.65) LR − 0.42 (0.28–0.64) Model 5 LR + 2.04 (1.49–2.81) LR − 0.39 (0.26–0.60)	Model 2 presented best performance. The results can inform identification of high-risk root caries individuals only in a caries-active population	Caries (coronal and root) prevalence at baseline 100% Caries increment (3 years) 49%
Sánchez-García, 2011 [23]	*N* = 6 *• Life- style situational* (dental mouthwash–oral hygiene routines, smoking) *• Physiological* (limitations in basic daily activities–general health) *• Oral biological* (ms ≥ 10^5^ CFU/ml saliva) *• Caries experience* (healthy root surfaces, root caries index)	Sens 0.16, Spec 0.98, PPV 0.67, NPV 0.81 AUC 0.75	LR + 7.09 (3.29–15.30) LR + 0.86 (0.80–0.93)	A good prediction model for 21-month root caries incidence in elderly (mean age 71.8; range 60–74)	Caries prevalence at baseline coronal caries 100% root caries 44% Caries increment (1 year) 22%
**Studies of model validation**					
* Coronal caries*					
Beck, 1992 [24]	Modified models of high-risk model Disney et al. [15] *N* = 20 Model 1 Any risk prediction model *• Societal structural* (education-head of household, referral caries score) *• Life-style situational* (tooth-brushing, F-tablets, dental visit last year–3 predictors) *• Physiological* (race) *• Oral biological* (ms in saliva, lbc in saliva) *• Tooth* (tooth morphology, sound permanent surfaces) *• Caries experience* (baseline dmfs, baseline DMFS, white spot lesions) *• Other predictors* (examiner agreement–4 predictors, predicted caries score) *N* = 13 Model 2 Any risk etiologic model *• life-style situational* (dental visit last year–2 predictors) *• Tooth* (tooth morphology, fluorosis, sound permanent surfaces, sealants) *• Oral biological* (ms in saliva, lbc in saliva, mean plaque score) *• Other predictors* (examiner agreement–4 predictors, predicted caries score)	Model 1 Any risk prediction model Aiken age 6.6 years Sens 0.80, Spec 0.61, PPV 0.73, NPV 0.69 Portland age 6.9 years Sens 0.66, Spec 0.78, PPV 0.57, NPV 0.84 Aiken age 10.7 years Sens 0.84, Spec 0.54, PPV 0.78, NPV 0.64 Portland age 10.8 years Sens 0.76, Spec 0.71, PPV 0.68, NPV 0.78 Model 2 Any risk etiologic model Aiken age 6.6 years Sens 0.74, Spec 0.55, PPV 0.68, NPV 0.62 Portland age 6.9 years Sens 0.59, Spec 0.74, PPV 0.51, NPV 0.80 Aiken age 10.7 years Sens 0.81, Spec 0.50, PPV 0.75, NPV 0.58 Portland age 10.8 years Sens 0.69, Spec 0.65, PPV 0.62, NPV 0.71	Model 1 Any risk prediction model Aiken age 6.6 years LR + 2.05 (1.82–2.31) LR − 0.33 (0.28–0.39) Portland age 6.9 years LR + 3.00 (2.57–3.50) LR − 0.44 (0.37–0.51) Aiken age 10.7 years LR + 1.83 (1.62–2.06) LR − 0.30 (0.24–0.36) Portland age 10.8 years LR + 2.62 (2.27–3.03) LR − 0.34 (0.28–0.40) Model 2 Any risk etiologic model Aiken age 6.6 years LR + 1.64 (1.47–1.84) LR − 0.47 (0.40–0.55) Portland age 6.9 years LR + 2.27 (1.95–2.64) LR − 0.55 (0.48–0.63) Aiken age 10.7 years LR + 1.62 (1.44–1.82) LR − 0.38 (0.31–0.46) Portland age 10.8 years LR + 1. 97 (1.73–2.25) LR − 0.48 (0.41–0.56)	Any risk etiologic models with fewer significant variables, appear to have similar, but slightly lower utility (sensitivity and specificity) compared to Any risk prediction models and appear to be more broadly applicable across populations	Caries prevalence at baseline NR Caries increment (3 years) Aiken 6.6 years 57% 10.7 years 66% Portland 6.9 years 31% 10.8 years 45% High-risk model identical with that presented by Disney et al. [15]
Birpou, 2019 [25]	*N* = 7–9 predictors in 8 modified Cariogram models with 1- and 2 years follow-up *Standard set* Model 1 Cariogram 1 *N* = 9 *• Life-style situational* (diet content, diet frequency, fluoride programme) *• Physiological* (related diseases) *• Oral biological* (ms in saliva with four thresholds, saliva buffer, plaque amount) *• Caries experience* (dmft with two thresholds) *• Other predictors* (clinical judgement) Model 2 Cariogram 2 *N* = 8 Model 1 excluding saliva buffer Model 3 Cariogram 3 *N* = 8 Model 1 excluding ms in saliva Model 4 Cariogram 4 *N* = 7 Model 1 excluding saliva buffer and ms in saliva *High risk set* Model 5 Cariogram 5 *N* = 9 Same predictors as Model 1 Model 6 Cariogram 6 *N* = 8 Model 1 excluding saliva buffer Model 7 Cariogram 7 *N* = 8 Model 1 excluding ms in saliva Model 8 Cariogram 8 *N* = 7 Model 1 excluding saliva buffer and ms in saliva	*Standard set* Model 1 Cariogram 1 1 year follow-up Sens 0.66, Spec 0.57, AUC 0.62 2 years follow-up Sens 0.68, Spec 0.59, AUC 0.65 Model 2 Cariogram 2 1 year follow-up Sens 0.65, Spec 0.57, AUC 0.62 2 years follow-up Sens 0.66, Spec 0.59, AUC 0.62 Model 3 Cariogram 3 1 year follow-up Sens 0.60, Spec 0.65, AUC 0.61 2 years follow-up Sens 0.64, Spec 0.68, AUC 0.65 Model 4 Cariogram 4 1 year follow-up Sens 0.56, Spec 0.65, AUC 0.61 2 years follow-up Sens 0.60, Spec 0.68, AUC 0.65 *High risk set* Model 5 Cariogram 5 1 year follow-up Sens 0.66, Spec 0.57, AUC 0.62 2 year follow-up Sens 0.68, Spec 0.58, AUC .65 Model 6 Cariogram 6 1 year follow-up Sens 0.65, Spec 0.65, AUC 0.62 2 years follow-up Sens 0.66, Spec 0.59, AUC 0.66 Model 7 Cariogram 7 1 year follow-up Sens 0.57, Spec 0.65, AUC 0.61 2 years follow-up Sens 0.61, Spec 0.68, AUC 0.65 Model 8 Cariogram 8 1 year follow-up Sens 0.56, Spec 0.65, AUC 0.62 2 years follow-up Sens 0.60, Spec 0.68, AUC .66	*Standard set* Model 1 Cariogram 1 1 year follow-up LR + 1.55 (1.13–2.13) LR − 0.59 (0.41–0.85) 2 years follow-up LR + 1.63 (1.17–2.26) LR − 0.55 (0.38–0.82) Model 2 Cariogram 2 1 year follow-up LR + 1.52 (1.11–2.09) LR − 0.61 (0.42–0.88) 2 years follow-up LR + 1.60 (1.15–2.22) LR − 0.58 (0.40–0.84) Model 3 Cariogram 3 1 year follow-up LR + 1.69 (1.17–2.44) LR − 0.62 (0.45–0.86) 2 years follow-up LR + 1.97 (1.33–2.90) LR- 0.54 (0.38–0.76) Model 4 Cariogram 4 1 year follow-up LR + 1.58 (1.09–2.30) LR − 0.68 (0.50–0.93) 2 years follow-up LR + 1.88 (1.24–2.74) LR − 0.60 (0.43–0.83) *High risk set* Model 5 Cariogram 5 1 year follow-up LR + 1.55 (1.13–2.13) LR − 0.59 (0.41–0.85) 2 years follow-up LR + 1.63 (1.17–2.26) LR − 0.55 (0.38–0.82) Model 6 Cariogram 6 1 year follow-up LR + 1.85 (1.29–2.65) LR − 0.54 (0.38–0.77) 2 years follow-up LR + 1.60 (1.15–2.22) LR − 0.58 (0.40–0.84) Model 7 Cariogram 7 1 year follow-up LR + 1.62 (1.12–2.35) LR − 0.66 (0.49–0.90) 2 years follow-up LR + 1.88 (1.27–2.79) LR − 0.58 (0.42–0.80) Model 8 Cariogram 8 1 year follow-up LR + 1.58 (1.09–2.30) LR − 0.68 (0.50–0.93) 2 years follow-up LR + 1.84 (1.24–2.74) LR − 0.60 (0.43–0.83)	Cariogram with various factors and settings displayed suboptimal ability to predict caries in this population (aged 2–5)	Caries prevalence at baseline 37% Caries increment 1 years 52% 2 years 53% Considering the high prevalence of caries increment it may have been expected that the high set would result in higher sensitivity and specificity than standard set
Campus, 2012 [26]	*N* = 7 Modified Cariogram *• Life-style situational* (diet content, diet frequency, fluoride programme) *• Physiological* (related diseases) *• Oral biological* (≥ 10^5^ ms in saliva, plaque amount) *• Caries experience* (cavity from 0–caries-free to 3 as sum of dmft and DMFS)	Sens 0.83, Spec 0.85, AUC 0.93	LR + 5.53 (4.36–7.03) LR − 0.2 (0.16–0.25)	Results showed high validity of Cariogram in schoolchildren	Data collected as lbc counts used as a measure of cariogenic diet Caries prevalence at baseline 29% Caries increment (2 years) 54%
Christian, 2020 [27]	*N* = 13 Modified CAMBRA *• Societal structure* (parent/caregiver: low socioeconomic status and/or low health literacy, mother/caregiver: decay-free last three years, mother/caregiver: xylitol chewing-gum/lozenges 2–4 × daily) *• Life-style situational* (developmental problems, no dental home/episodic dental care, dental home and regular dental care, frequent between-meal snacks of sugars/cooked starch/sugared beverages, continually uses bottle—contains fluids other than water, sleeps with a bottle or nurses on demand, lives in a fluoridated community, or takes fluoride supplements, fluoridated toothpaste daily) *• Oral biological* (obvious plaque on teeth and/or gums bleed easily)	Follow-up 1.5 years Sens 0.74, Spec 0.35, PPV 0.20, NPV 0.86, AUC close to 0.5 Follow-up 2.5 years Sens 0.70, Spec 0.36¸ PPV 0.37, NPV 0.69, AUC close to 0.5	Follow-up 1.5 years LR + 1.14 (0.92–1.41), LR − 0.74 (0.42–1.31) Follow-up 2.5 years LR + 1.09 (0.90–1.33), LR − 0.83 (0.55–1.26)	CAMBRA in its current form may not be ideal for use in risk-based disease management among young Victorian children. Due to its low specificity, it is highly likely that the use of this risk assessment tool could be driving over-treatment	Caries prevalence at baseline 0% Caries increment 1.5 years 18% 2.5 years 35%
Dolic, 2020 [28]	*N* = 9 Cariogram *• Life-style situational* (diet content, diet frequency, fluoride programme) *• Physiological* (related diseases) *• Oral biological* (ms in saliva, saliva secretion, saliva buffer, plaque amount) *• Caries experience* (cavity)	Threshold 1 (moderate, low, and very low risk) Sens 0.54, Spec 0.69, PPV 0.78, NPV 0.42 LR + 1.47, LR − 0.66 Threshold 2 (moderate, high, and very high risk) Sens 0.81, Spec 0.58, PPV 0.80, NPV 0.60 LR + 1.92, LR − 0.32	Threshold 1 (moderate, low, and very low risk) LR + 1.74 (0.93–3.25) LR − 0.67 (0.45–0.98) Threshold 2 (moderate, high, and very high risk) LR + 1.93 (1.21–3.09) LR − 0.33 (0.17–0.62)	Cariogram can be a useful tool for caries prediction. It is valid and highly predictive	Data collected as lbc counts (Dentocult) used as a measure of cariogenic diet Caries prevalence at baseline NR Caries increment NR No report of thresholds for ms, lbc, or caries experience
Gao, 2013 [29]	*N* = 5–14 in 10 different models Model 1 NUS-CRA Comprehensive *N* = 11 *• Societal structure* (family socioeconomic status) *• Life-style situational* (infant feeding history, diet, fluoride. plaque index) *• Physiological* (age, ethnicity, systemic health) *• Oral biological* (ms in saliva, lbc in saliva) *• Caries experience* (cavity) Model 2 NUS-CRA screening *N* = 9 Model 1 excluding ms and lbc in saliva Model 3 Cariogram Comprehensive *N* = 9 predictors see study below by Hänsel Petersson et al. [30] Model 4 Cariogram screening *N* = 5 Model 3 excluding saliva secretion, saliva buffer, ms in saliva, lbc in saliva Model 5 CAT Comprehensive I *N* = 12 *• Societal structural* (family socioeconomic status, dental attendance) *• Life-style situational* (diet, fluoride, oral hygiene, dental appliance) *• Physiological* (systemic health) *• Oral biological* (ms in saliva, saliva secretion) *• Caries experience* (past caries, white spot lesion, enamel defects) Model 6 CAT comprehensive II *N* = 11 Model 5 without family socioeconomic status Model 7 CAT screening I *N* = 10 Model 5 excluding saliva secretion and ms in saliva Model 8 CAT screening II *N* = 9 Model 7 without socioeconomic status Model 9 CAMBRA Comprehensive *N* = 14 *• Societal structural* (family socioeconomic status) *• Life-style situational* (infant feeding history, diet, fluoride, oral hygiene, dental appliance, dental attendance) *• Physiological* (systemic health, medication) *• Oral biological (*ms in saliva, lbc in saliva, saliva secretion) *• Caries experience* (past caries, white spot lesion) Model 10 CAMBRA Screening *N* = 11 Model 9 excluding ms in saliva, lbc in saliva, saliva secretion	Model 1 NUS-CRA comprehensive Sens 0.78, Spec 0.85, AUC 0.88 Model 2 NUS-CRA screening Sens 0.74, Spec 0.88, AUC 0.85 Model 3 Cariogram comprehensive Sens 0.65, Spec 0.79, AUC 0.78 Model 4 Cariogram screening Sens 0.63, Spec 0.78, AUC 0.76 Model 5 CAT comprehensive I Sens 1, Spec 0.04 Model 6 CAT comprehensive II Sens 0.99, Spec 0.04 Model 7 CAT creening I Sens 0.98, Spec 0.05 Model 8 CAT screening II Sens 0.98, Spec 0.05 Model 9 CAMBRA comprehensive Threshold ≥ Moderate Sens 0.92, Spec 0.40 Threshold ≥ High Sens 0.84, Spec 0.63 Model 10 CAMBRA Screening Threshold ≥ Moderate Sens 0.98, Spec 0.20 Threshold ≥ High Sens 0.94, Spec 0.44	Model 1 NUS-CRA comprehensive LR + 5.31 (4.01–7.03) LR − 0.26 (0.19–0.34) Model 2 NUS-CRA screening LR + 4.81 (3.64–6.35) LR − 0.31 (0.24–0.40) Model 3 Cariogram comprehensive LR + 3.0 (2.36–3.82) LR − 0.45 (0.37–0.55) Model 4 Cariogram screening LR + 2.85 (2.24–3.61) LR − 0.48 (0.39–0.58) Model 5 CAT comprehensive I LR + 1.04 (1.02–1.06) LR − 0 Model 6 CAT comprehensive II LR + 1.03, (1.01–1.06) LR − 0.15 (0.02–1.10) Model 7 CAT screening I LR + 1.04 (1.01–1.08) LR − 0.21 (0.05–0.92) Model 8 CAT Screening II LR + 1.04 (1.0–1.08) LR − 0.31 (0.09–1.04) Model 9 CAMBRA Comprehensive Threshold ≥ Moderate LR + 1.52 (1.37–1.68) LR − 0.21 (0.13–0.35) Threshold ≥ High LR + 2.26 (1.92–2.65) LR − 0.26 ( 0.18–0.37) Model 10 CAMBRA Screening Threshold ≥ Moderate LR + 1.22 (1.15–1.30) LR- 0.14 (0.06–0.33) Threshold ≥ High LR + 1.66 (1.50–1.86) LR − 0.14 (0.08–0.26)	Our findings supported algorithm modelling. NUS-CRA appeared to be a baseline useful program with sufficient sensitivity and specificity in Hong Kong children	Caries prevalence at baseline 35% Caries increment (1 year) 37%
Hänsel Petersson, 2015 [30]	*N* = 9 Cariogram *• Life-style situational* (diet content, diet frequency, fluoride programme) *• Physiological* (related diseases) *• Oral biological* (ms in saliva with four thresholds, saliva secretion, saliva buffer, plaque amount) *• Caries experience* (DMFT cavity with four thresholds)	Threshold % DFS new lesions 80% Sens 0.89, Spec 0.34 PPV 0.42, NPV 0.85 60% Sens 0.61, Spec 0.71 PPV 0.53, NPV 0.77 40% Sens 0.26, Spec 0.91 PPV 0.60, NPV 0.69 20% Sens 0.12, Spec 0.95 PPV 0.55, NPV 0.67	Threshold % DFS new lesions 80% LR + 1.34 (1.26–1.43) LR − 0.32 (0.23–0.45) 60% LR + 2.11 (1.82–2.45) LR − 0.55 (0.47–0.63) 40% LR + 2.72 (2,02–3.68) LR − 0.82 (0.77–0.88) 20% LR + 2.29 (1.48–3.55) LR − 0.93 (0.89–0.97)	Cariogram did not perform better than a risk assessment scheme based on past Caries experience and caries progression, over a 3-year period in young adults	Data collected as lbc counts used as a measure of cariogenic diet Caries prevalence at baseline 77% Caries increment (3 years) 35%
Hänsel Petersson, 2010 [31]	Model 1 Cariogram *N* = 9 *• Life-style situational* (diet content, diet frequency, fluoride program) *• Physiological* (related diseases) *• Oral biological* (ms in saliva with four thresholds, saliva secretion, saliva buffer, plaque amount) *• Caries experience* (dentin caries DMFS with four thresholds) Model 2 *N* = 8 Model 1 excluding ms in saliva Model 3 *N* = 8 Model 1 excluding saliva buffer Model 4 *N* = 8 Model 1 excluding saliva secretion Model 5 *N* = 6 Model 1 excluding ms in saliva, saliva buffer, saliva secretion	Model 1 Sens 0.73, Spec 0.60 PPV 0.45, NPV 0.83, LR + 1.80, LR- 0.45 AUC 0.75 Model 2 Sens 0.84, Spec 0.47 PPV 0.41, NPV 0.86, LR + 1.60, LR − 0.36 AUC 0.73 Model 3 Sens 0.79, Spec 0.51 PPV 0.42, NPV 0.85, LR + 1.60, LR − 0.41 AUC 0.75 Model 4 Sens 0.77, Spec 0.49 PPV 0.41, NPV 0.83, LR + 1.50, LR − 0.45 AUC 0.75 Model 5 Sens 0.90, Spec 0.20 PPV 0.34, NPV 0.82, LR + 1.10, LR − 0.50 AUC 0.72	Model 1 LR + 1.83, (1.52–2.19) LR − 0.45 (0.33–0.61) Model 2 LR + 1.58 (1.38–1.82) LR − 0.34 (0.22–0.52) Model 3 LR + 1.61 (1.38–1.88) LR − 0.41 (0.29–0.59) Model 4 LR + 1.51 (1.30–1.76) LR − 0.47 (0.33–0.66) Model 5 LR + 1.13 (1.03–1.22) LR − 0.50 (0.28–0.90)	Accuracy of caries prediction in school children was significantly impaired when Cariogram was applied without enumeration of salivary tests. ms enumeration seemed to be most important of the salivary variables	Data collected as lbc counts used as a measure of cariogenic diet Caries prevalence at baseline 40% Caries increment (2 years) 31%
Holgerson, 2009 [32]	*N* = 7 Modified Cariogram *• Life-style situational* (diet frequency, oral hygiene, fluorides) *• Physiological* (related diseases) *• Oral biological* (ms in saliva—counts with four thresholds) *• Caries experience* (above average for age group) *• Other predictors* (clinical judgement)	Sens 0.46, Spec 0.88 PPV 0.90, NPV 0.40, LR + 3.7, LR − 0.6	LR + 3.83 (1.39–10.58) LR − 0.61 (0.40–0.94)	Modified Cariogram in 2-year-old children resulted in high sensitivity for future caries, but the method lacked accuracy and precision	Caries prevalence at baseline 3% Caries increment (5 years) 71% Clinical judgement added after result of Cariogram was obtained
Pang, 2021 [19]	*N* = 7 Predictors described below Pang 2021 in studies of model development described above	Total AUC 0.73 (0.68–0.79) Low caries risk (DMFT ≥ 1 caries lesion) Sens 0.29, Spec 0.63, PPV 0.29, NPV 0.63 Moderate caries risk Sens 0.34, Spec 0.66, PPV 0.48, NPV 0.52 High caries risk Sens 0.59, Spec 0.68, PPV 0.74, NPV 0.53 Very high caries risk Sens 0.66, Spec 0.58, PPV 0.90, NPV 0.22	Low caries risk LR + 0.78 (0.57–1.08) LR − 1.13 (0.96–1.33) Moderate caries risk LR + 1.00 (0.73–1.38) LR − 1.00 (0.85–1.18) High caries risk LR + 1.86 (1.39–2.48) LR- 0.60 (0.49–0.74) Very high caries risk LR + 1.54 (1.22–1.94) LR − 0.60 (0.47–0.76)	This caries risk prediction model can accurately identify a high-risk population. The model can be utilized as a powerful tool at community level	Sample different for studies of model development and model validation Caries prevalence at baseline 40% Caries increment (1.7 years) 63% ICDAS codes 3–6, Pitts and Ekstrand, [35]
* Root caries*					
Hayes, 2017 [33]	*N* = 9 Model 1 Cariogram *• Life-style situational* (diet content, diet frequency, fluoride programme) *• Physiological* (related diseases) *• Oral biological* (ms ≥ 10^5^ counts per ml saliva, saliva secretion, saliva buffer, plaque amount) *• Caries experience* (normal caries experience: mean DMFT score 22.4 ± 5.3 range 17–28) Model 2 *N* = 8 Model 1 excluding ms in saliva Model 3 *N* = 8 Model 1 excluding saliva buffer Model 4 *N* = 8 Model 1 excluding saliva secretion Model 5 *N* = 6 Model 1 excluding ms in saliva, saliva buffer, saliva secretion	Model 1 Cariogram Sens 0.79, Spec 0.63, PPV 0.40, NPV 0.90 AUC 0.77 Model 2 Model 1 excluding ms Sens 0.74, Spec 0.73, PPV 0.48, NPV 0.90 AUC 0.80 Model 3 Model 1 excluding saliva buffer Sens 0.79, Spec 0.58, PPV 0.39, NPV 0.89 AUC 0.76 Model 4 Model 1 excluding saliva secretion Sens 0.79, Spec 0.58, PPV 0.39, NPV 0.89 AUC 0.77 Model 5 Model 1 excluding ms, saliva buffer, saliva secretion Sens 0.73, Spec 0.66, PPV 0.42, NPV 0.88 AUC 0.79	Model 1 Cariogram LR + 2.12 (1.71–2.63) LR − 0.34 (0.21–0.54) Model 2 Model 1 excluding ms LR + 2.78 (2.14–3.62) LR − 0.35 (0.23–0.52) Model 3 Model 1 excluding saliva buffer LR + 1.88 (1.53–2.29) LR − 0.37 (0.23–0.59) Model 4 Model 1 excluding saliva secretion LR + 1.88 (1.53–2.29) LR − 0.37 (0.23–0.59) Model 5 Model 1 excluding ms, saliva buffer, saliva secretion LR + 2.13 (1.68–2.69) LR − 0.41 (0.28–0.61)	Cariogram may be clinically useful in determining future root caries in independently living older dentate adults (aged ˃ 65)	Data collected as lbc counts used as a measure of cariogenic diet Caries prevalence at baseline NR Caries increment (2 years) 25%

*Abbreviations*: *ms* mutans streptococci, *lbc* lactobacilli, *DMFT* decayed missing filled teeth (permanent), *DMFS* decayed missing filled surfaces (permanent), *DS* decayed surfaces (permanent), *dmft* decayed missing filled teeth (primary), *dmfs* decayed missing filled surfaces (primary), *saliva buffer* saliva buffering capacity, *saliva secretion* saliva secretion flow rate, *NR* not reported

### Risk of bias and concern regarding applicability

Pairs of review authors independently assessed risk of bias (ROB) and concern regarding applicability using PROBAST [5, 6] with 20 signalling questions in 4 domains for ROB (participants, predictors, outcome, analysis) and 3 domains for applicability (participants, predictors, outcome). Each signalling question is answered by yes, probably yes, no, probably no, or no information [36]. Based on the ratings, the global ROB and applicability concerns are judged as low, high, or unclear [6]. Disagreements were resolved by discussion between 4 review authors.

### Analysis of predictors and model performance

Candidate predictors in developmental model that expressed similar characteristics were grouped in categories, and allocated to different levels of a model for a caries process. The performance of each model was re-calculated as estimated LRs based on the sensitivity and specificity presented in included studies: LR + equals sensitivity divided by (1—specificity) and LR − equals (1—sensitivity) divided by specificity. We considered the analysed models to be useful for prediction of caries increment when LRs + were ≥ 5.0 and conversely, ruling out caries increment when LRs − were ≤ 0.20 [37]. Confidence intervals for LRs were calculated using the method described by Koopman [38]. We intended to perform meta-analyses by pooling estimates of the LRs whenever 3 or more studies were based on similar prediction model, participant age group and definition of caries increment, but due to variation across studies, this was waived.

## Results

### Study selection

**Fig. 1:**
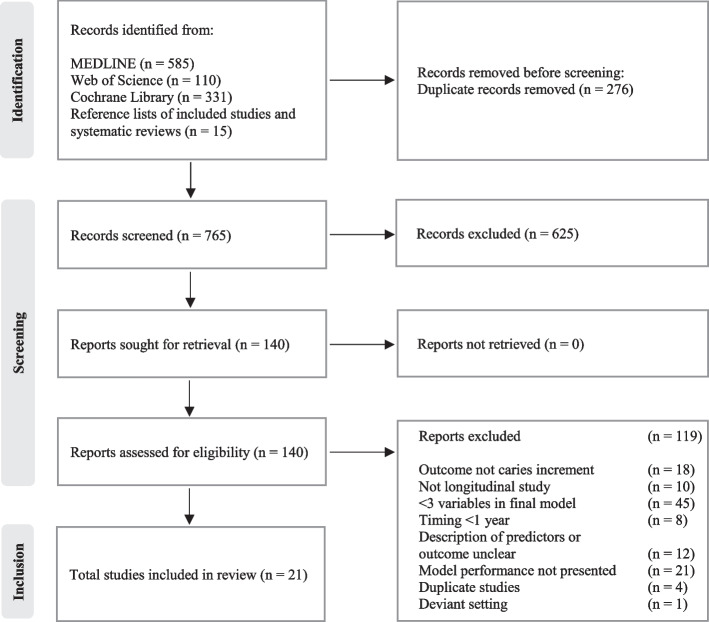
PRISMA flow chart for study selection

Figure 1 shows the flow of records identified through the searches and study selection. Out of 140 full-text publications, 21 were included. Excluded full-text publications with reasons for exclusion are provided in Additional file 3.

### Study characteristics

The main characteristics of the studies included are presented in Table 1 and Additional file 4. References to publications describing methodology for candidate predictors are listed in Additional file 5. In 11 studies [13–23], model development was emphasized (8 studies with 23 models of coronal caries, 2 studies with 6 models of root caries, one study with a model of coronal and root caries). Ten studies [19, 24–33] focused on model validation (9 studies with 31 models of coronal caries and one study with 5 models of root caries). Most validation studies were not performed according to the study describing the model development. We did not find original studies presenting model development of CAMBRA (Caries Management by Risk Assessment) or CAT (Caries-risk Assessment Tool) with model performance measures according to CHARMS. All studies were cohort studies except for two studies, which were described as case–control and cluster sample studies, respectively.

There was high inter-study variability in predictors, outcome definitions and timing of outcome measurements. Five studies used bite-wing radiography (Table 1) and enamel caries was included in the outcome in only 2 studies. Regarding participants, they were generally children or adolescents (aged 2–19) in studies of coronal caries, and adults (aged 52–80) in studies of root caries. Sample sizes ranged from 21 to 1576 participants. In studies of model development, the number of events (outcomes) in relation to the number of variables, i.e., candidate predictors (events per variable = EPV) varied between 0.72 and 114.8, being ≥ 20 in 1 study, ≥ 10 in 2 studies, and < 10 in the remaining studies (Table 1). In half of the model validation studies, the number of events and non-events was in excess of 100 (Table 1). Logistic regression analysis was the most prevalent modelling technique, using univariate analyses to filter potential predictors for the final model. Algorithm-based modelling was used in 2 studies and a machine learning approach in 1 study.

Reported model performances are presented in Table 2. Sensitivity and specificity were reported in all studies, AUC was reported in 6 studies of model development and 12 studies of model validation, and LRs were reported in 3 studies of model validation. One study presented calibration. Confidence intervals were reported in 4 studies.

### Risk of bias (ROB) and concern regarding applicability

**Fig. 2:**
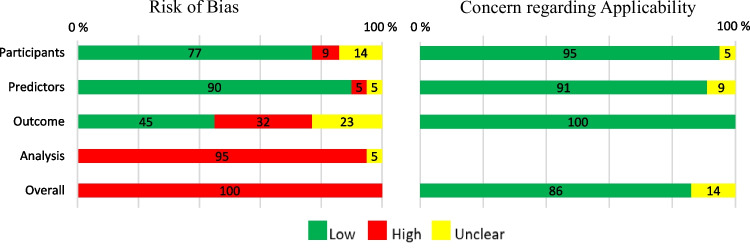
Distribution of risk of bias and concern regarding applicability for each domain and overall

The distribution of ROB and applicability for each domain and overall is presented in Fig. 2. Overall, ROB was high; in the *Analysis* domain all but one study and in the *Outcome* domain one third of the studies showed high ROB, while in the *Participant* and *Predictor* domains the ROB was low. Concern regarding applicability was rated low in 86% of the studies.

ROB and concern regarding applicability of each study are presented in Table 3 and detailed information on signalling question responses is found in Additional file 6. The *Participant* domain was assessed at high or unclear ROB in 5 studies since inclusion or exclusion criteria were missing or unclear. Although the *Predictor* domain was assessed at low ROB in most studies, there was considerable uncertainty regarding the thresholds and measurements. For the *Outcome* domain, half of the studies showed high or unclear ROB. No estimates of measurement error of the method determining the outcome were presented. Only 1 study described that the outcome was determined without knowledge of predictor information. In the *Analysis* domain*,* high ROB was usually assigned due to insufficient number of EPV in model development studies or number of events in model validation studies. Other frequent reasons were inappropriate handling (or no information) of continuous and categorical predictors, and selection of predictors based on univariate analysis in model development studies. Regarding applicability, concern was low for all but 3 studies; 1 study was rated as unclear regarding the domain *Participants* and 2 regarding the domain *Predictors* (Table 3)*.*

**Table 3:** Risk of bias and concern regarding applicability for studies of multivariable prediction models of caries increment

Study First author, year of publication [reference]	ROB^a^				Concern regarding applicability^a^			Overall^b^	
	Participants	Predictors	Outcomes	Analysis	Participants	Predictors	Outcomes	ROB	Applicability
*Studies of model development*									
Angulo, 1995 [13]	** − **	** + **	** − **	** − **	**?**	** + **	** + **	** − **	**?**
Demers, 1992 [14]	** + **	** + **	** + **	** − **	** + **	** + **	** + **	** − **	** + **
Disney, 1992 [15]	** + **	** + **	** + **	** − **	** + **	** + **	** + **	** − **	** + **
Fontana, 2011 [16]	** + **	** + **	** + **	** − **	** + **	** + **	** + **	** − **	** + **
Gao, 2010 [17]	** + **	** + **	** + **	** − **	** + **	** + **	** + **	** − **	** + **
Hänsel Petersson, 2002 [18]	** + **	** + **	**?**	** − **	** + **	** + **	** + **	** − **	** + **
Pang, 2021 [19]	**?**	** + **	** − **	** − **	** + **	** + **	** + **	** − **	** + **
Powell, 1991 [21]	**?**	**?**	** − **	** − **	** + **	**?**	** + **	** − **	**?**
Ritter, 2016 [22]	** + **	** − **	**?**	** − **	** + **	** + **	** + **	** − **	** + **
Sánchez-García, 2011 [23]	** + **	** + **	** + **	** − **	** + **	** + **	** + **	** − **	** + **
Sánchez-Pérez, 2009 [20]	** + **	** + **	**?**	** − **	** + **	** + **	** + **	** − **	** + **
*Studies of model validation*									
Beck, 1992 [24]	** + **	** + **	**?**	** − **	** + **	** + **	** + **	** − **	** + **
Birpou, 2019 [25]	** + **	** + **	** − **	** − **	** + **	** + **	** + **	** − **	** + **
Campus, 2012 [26]	** + **	** + **	** + **	** − **	** + **	** + **	** + **	** − **	** + **
Christian, 2020 [27]	** + **	** + **	** − **	** − **	** + **	** + **	** + **	** − **	** + **
Dolic, 2020 [28]	** − **	** + **	** + **	** − **	** + **	** + **	** + **	** − **	** + **
Gao, 2013 [29]	** + **	** + **	** + **	** − **	** + **	** + **	** + **	** − **	** + **
Hayes, 2017 [33]	** + **	** + **	** + **	** − **	** + **	** + **	** + **	** − **	** + **
Hänsel Petersson, 2015 [30]	** + **	** + **	**?**	** − **	** + **	** + **	** + **	** − **	** + **
Hänsel Petersson, 2010 [31]	** + **	** + **	** + **	** − **	** + **	** + **	** + **	** − **	** + **
Holgerson, 2009 [32]	** + **	** + **	** − **	** − **	** + **	** + **	** + **	** − **	** + **
Pang, 2021 [19]	**?**	** + **	** − **	**?**	** + **	**?**	** + **	** − **	**?**

Assessment according to PROBAST [6]: “ + ” indicates low ROB or low concern regarding applicability; “ − ” indicates high ROB or high concern regarding applicability; “?” indicates unclear ROB or unclear concern regarding applicability

^a^Each domain of ROB/concern regarding applicability is based on responses of respective items (Additional file 6) as follows: if all items are answered with “yes”, the domain is at low ROB/concern regarding applicability. If in at least one item, the response is “unclear” and the rest of the items are “Yes”, the ROB/concern regarding applicability is unclear. If the response is “no” in at least one item, regardless of other item responses the domain is at high ROB/concern regarding applicability

^b^Overall assessment is expressed as follows: low ROB/concern regarding applicability if all domains are assessed low ROB/concern regarding applicability; high ROB/concern regarding applicability in case at least one domain is assessed high ROB/concern regarding applicability; if the risk is unclear in at least one domain and all other domains are low ROB/concern regarding applicability, final assessment remains unclear

*Abbreviations*: *ROB* risk of bias, *PROBAST* Prediction model Risk Of Bias ASsessment Tool

### Analysis of predictors and model performance

**Fig. 3:**
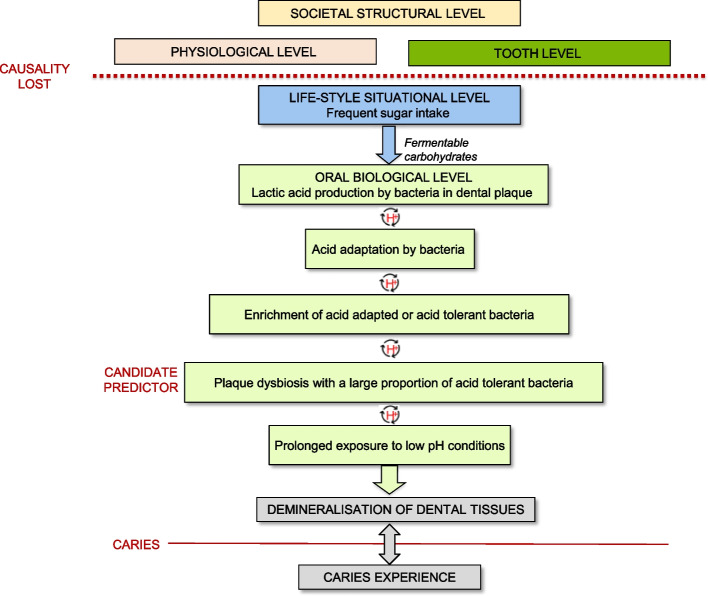
A caries process model with 6 levels. Each level holds a set of variables used as predictors in included studies. The horizontal line CAUSALITY LOST indicates that the 3 top levels do not commute with the other levels. Protons in dental plaque (H^+^) are the driving force in the caries process in development of dysbiosis as well as demineralisation of dental tissues. Acid tolerant bacteria in dental plaque are proposed as novel candidate predictors

Based on a caries process model (Fig. 3), we allocated the predictors to the following levels: (i) societal structural, (ii) physiological, (iii) tooth, (iv) life-style situational, (v) oral biological, (vi) caries experience and other types of predictors. In the following text, variables considered in the model development are labelled *candidate predictors* and variables included in the final models, *predictors* in accordance with CHARMS.

#### Predictors in studies of model development

Sampling methods, measurement methods, and thresholds varied across studies. For example, caries experience and caries increment were assessed using different criteria (e.g., according to WHO, Radike, ICDAS [34, 35, 39–43]) and caries was defined as dentinal caries or cavity in all but 2 studies that included enamel lesions. Predictors at the Societal structural Level were collected using unvalidated questionnaires. One example of methods not clearly reported was for the predictor mutans streptococci: information on detection limits in saliva was not given, no criteria for colony forming units on Mitis-Salivarius Bacitracin (MSB) agar was offered, and biochemical testing were not used to confirm mutans streptococci.

**Fig. 4:**
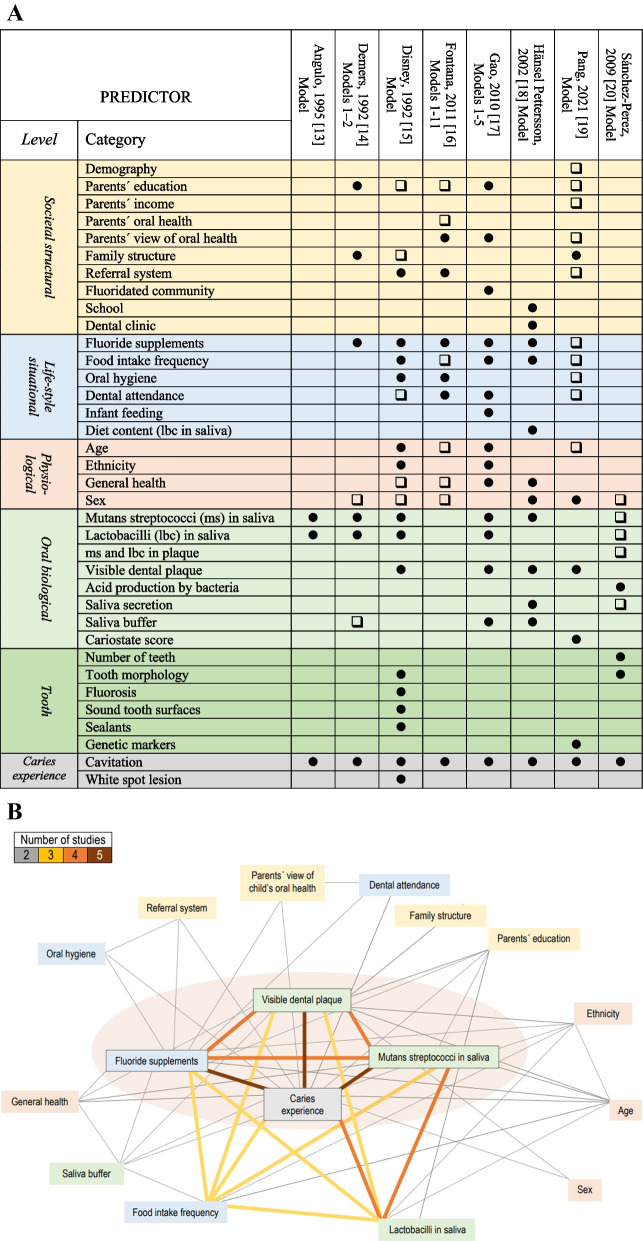
Predictors in developmental models of coronal caries increment. **A** Candidate predictor [□] and predictor remaining [●] in at least one final model of actual study. **B** Predictor networks in final models with coloured lines showing predictors included in 2–5 studies. The pink area indicates a set of 4 predictors identified in 4 studies

Altogether, more than 150 candidate predictors were identified, and the number included in each model ranged between 3 and 46 (Additional file 7). Many of these were similar in nature but their names varied across studies, e.g., food intake frequency was described with 21 different names. In studies of coronal caries, candidate predictors from 2 to 6 levels were represented, with 5 of them being the most prevalent (Fig. 4A). Final models of coronal caries included 31 predictors with between 3 and 23 predictors in each model and models of root caries included 16 predictors with between 6 and 13 predictors in each model. Three studies of coronal caries included ≥ 2 models and for those studies the information about predictors was merged in Fig. 4A. Caries experience was utilized as predictor in all studies; other commonly included predictors were visible dental plaque, mutans streptococci in saliva, and fluoride supplements (Fig. 4A). Predictor combinations (occurring in ≥ 2 studies) are illustrated as a network in Fig. 4B. The most prevalent set of 4 predictors was caries experience, use of fluoride supplements, mutans streptococci in saliva, and visible dental plaque (Fig. 4B), identified in 4 studies.

#### Performance of development and validation models

**Fig. 5:**
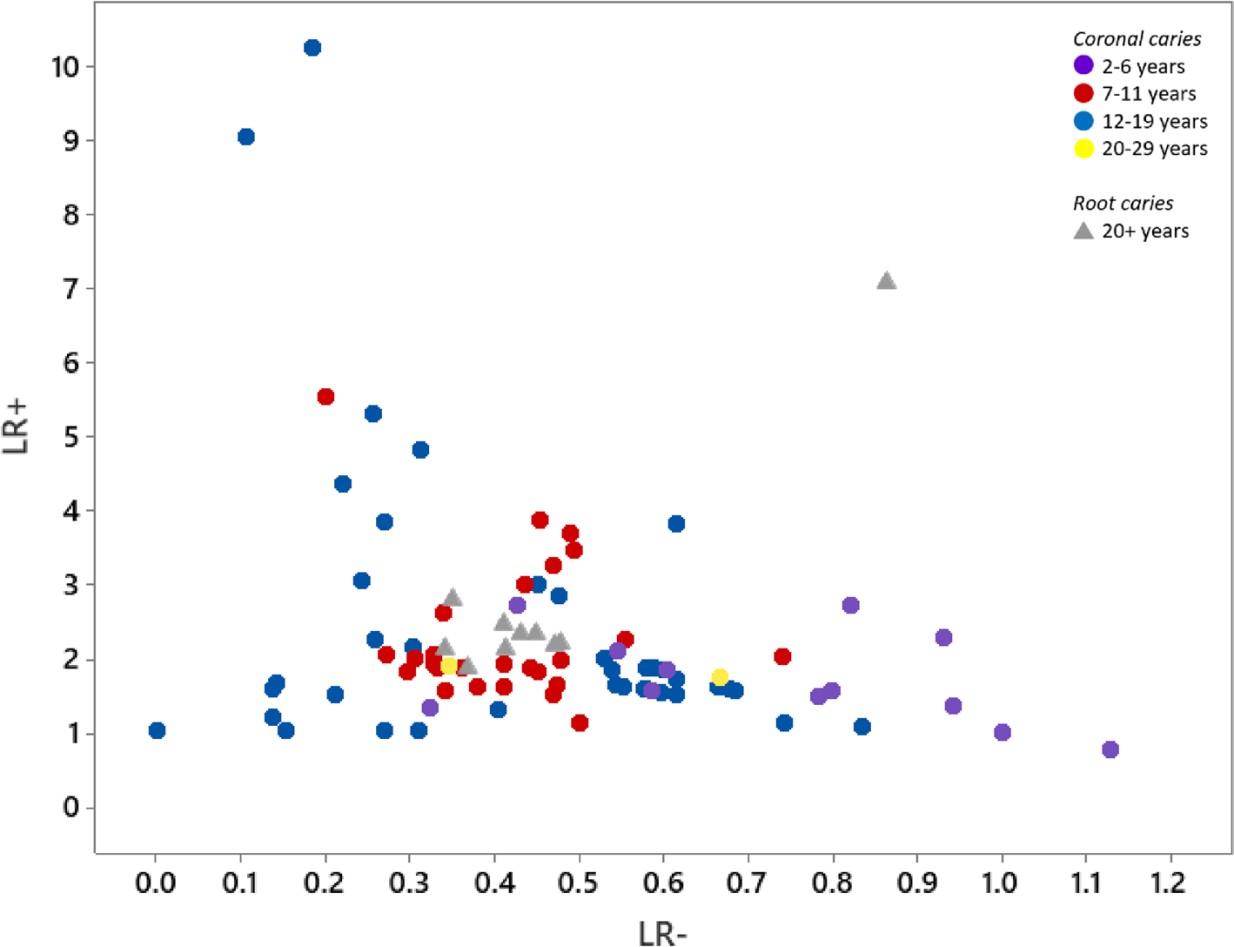
Positive (LR +) and negative likelihood ratio (LR −) of final models of caries increment for various age-groups

Owing to the heterogeneity of the studies and the high overall ROB, model performances are reported without meta-analyses, thus avoiding apparent estimates at odds with the underlying data. Table 2 presents model performances expressed as LRs. LR + ranged between 0.78 and 10.3 and LR − between 0.0 and 1.1. Models based on many predictors performed no better than models based on fewer predictors. For example, LR + was 3.5 and LR − 0.49 for the model with the highest number of predictors (*n* = 23), while a model with 6 predictors yielded LR + 10.3 and LR − 0.18. As shown in Fig. 5, LR +  ≥ 5 was achieved in 5 models, 4 of coronal caries in children [17, 26, 29], and 1 of root caries in the elderly [23]. LR −  ≤ 0.20 was expressed in 3 of these 5 models [17, 26, 29] and in 5 additional models [14, 21, 29]. Two models of children aged 3–6 differed in that 1 model included 12 predictors and the other model only 6 [17]. The model with 6 predictors achieved a somewhat higher LR + (10.3 *vs*. 9.0) but did not include the predictors fluorides and caries experience. The distribution of LRs related to age groups was scattered, further indicating heterogeneity (Fig. 5). For children aged 2–6 and adolescents aged 12–19, most LRs were scattered, whilst the LRs for schoolchildren aged 7–11 were more coherent.

#### Model validation of the Cariogram

**Fig. 6:**
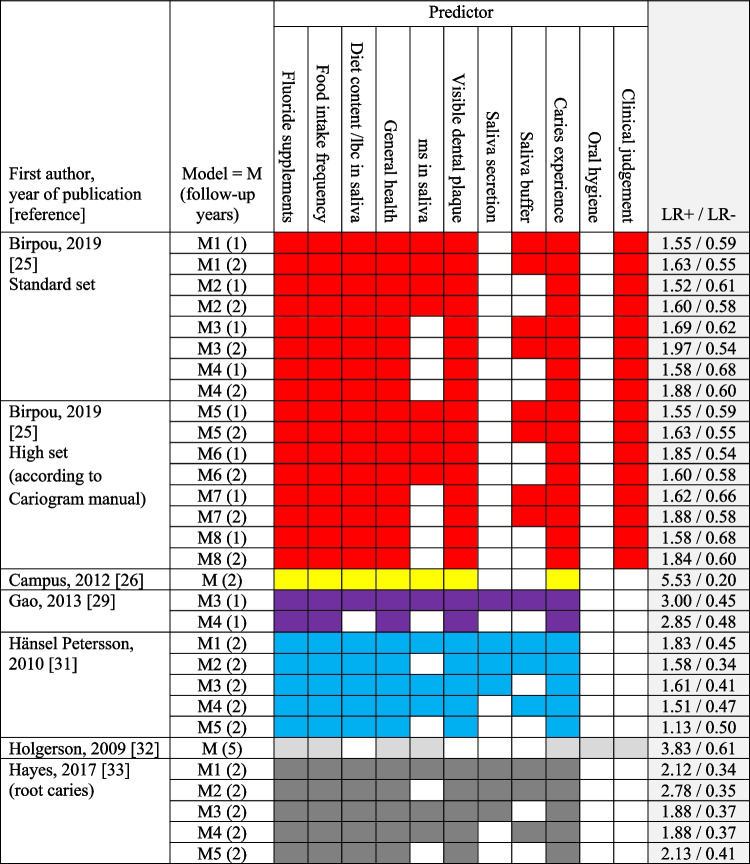
Predictors and positive (LR +) and negative likelihood ratio (LR −) in studies of model validation of Cariogram. lbc = lactobacilli; ms = mutans streptococci

Six studies of model validation (5 regarding coronal caries and 1 root caries) referred to the Cariogram. However, the studies did not validate the original Cariogram model [18] per se, but presented modifications of which. As shown in Fig. 6, models provided modest LR + (range 1.1–3.8) and LR − (range 0.5–0.61), with the exception of 1 model. LRs were not substantially influenced by the exclusion of the predictor mutans streptococci in saliva. In a study of root caries, LR + increased and LR − remained unchanged when the predictor mutans streptococci in saliva was omitted. Similarly, model performance was unaffected by removal of the predictors saliva secretion and saliva buffer.

## Discussion

### Main findings

In this SR of multivariable models of caries increment, we identified and critically appraised 11 studies of model development [13–23], and 10 of model validation [19, 24–33]. Model performance expressed as LR + of at least 5, a commonly used arbitrary definition for moderate increase in the probability of a condition after model implementation [44], was achieved for few models. All studies were appraised to have high ROB, in particular in the domain *Analysis*. Heterogeneity across the studies ruled out meta-analyses and thereby any conclusion about evidence for the applicability of caries prediction models included.

### Strengths and limitations

To the best of our knowledge, this is among the first systematic reviews of studies of model development and model validation of prediction of caries increment that applied CHARMS together with PROBAST. The strength of CHARMS is the thorough description of domains and key items relevant to extract with rationales, enabling reviewers and readers to understand the reasons for the items extracted. Even so, relevant data were sometimes difficult to identify since different terms for participants, predictors, outcomes, model development, and performance were used, and not always reported. While CHARMS was relied on to organize and identify relevant items, PROBAST was applied to identify potential sources of bias and concern regarding applicability. For the reporting of studies developing or validating prediction models, the TRIPOD Statement (Transparent Reporting of a multivariable prediction model for Individual Prognosis Or Diagnosis) [45] provides helpful.

As emphasized in a recent systematic review of oral health prediction models [46], there is a need to employ the same rigour to prediction models in dental as well as medical research.

Another strength of this review is the rigorous process by which 2 teams of 2 review authors independently screened records and selected full-text publications using protocols. Multiple rounds of piloting were used to refine the CHARMS and PROBAST protocols and we profited from being experts in different fields. As emphasized by Lasserson et al. [47], research teams with different expertise may identify different sources of evidence and reach different judgements. Additionally, the review authors were calibrated how to use the CHARMS and PROBAST tools.

Although the findings of this review are valuable and substantially add to the current literature, the study has limitations. We did not perform a search of grey literature, which could make the search more comprehensive. Another potential limitation is failing to assess publication bias.

### Critical appraisal using PROBAST

All studies were found to have high ROB, indicating that all models’ ability to predict caries increment is potentially flawed. In particular, concerns about the methods and inherent measurement errors were identified in the domains *Predictors* and *Outcomes*. Risk of bias is higher for predictors and outcomes that involve subjective judgment, such as the visual-tactile examination of caries used in the majority of included studies. Furthermore, the sensitivity is rather modest for visual examination to identify dental cavities and ranges from 0.12 to 0.50 depending on the raters [48]. This affects estimates of the predictor as well as the outcome (and thereby the predictive performance) but this limitation was not discussed in any study. Rater reliability for assessment of caries experience was sometimes reported, but this measure does not encompass the total measurement error.

High ROB was mainly found in the domain *Analysis*. In studies of model development, the EPV number was low and was not reported, but had to be calculated from other study information. EPV is generally poorly reported in prediction model studies [4]. To minimize overfitting, an EPV of at least 10 in model development studies has been widely recommended, but higher EPV (≥ 20) has also been suggested [6]. Only 1 study [17] in the current review was based on EPV > 20, and another 2 on EPV ≥ 10 [14, 18].

A second concern was that univariate analysis was used as selection method for the predictors in the final models. Significant association in univariate analysis is not recommended to merit inclusion due to risk of bias in 2 directions. Firstly, predictors may have a large but non-causal association with the outcome, and secondly, in small samples, predictors may only show association with the outcome after adjustment for other variables.

A final concern was model performance measures; in general, only the classification measures sensitivity and specificity were presented. Calibration was carried out in only 1 study [19]. In addition, most studies did not report statistical uncertainty even though *post facto* calculated confidence intervals for the LRs were wide for many models, i.e., a clear indication of low precision.

The high ROB identified in all studies of the current review is in accord with those reported by Du et al. [46] but differed from the results by Su et al. [49], who reported low ROB for 3 validation studies of coronal caries [26, 29, 31], which we rated as high ROB. Since no responses to the signalling questions as required by PROBAST were provided by Su et al. [49], a comparison of the conflicting results was untenable. Our results on high ROB of studies of prediction models, in particular in the domain *Analysis* were not exceptional. A meta-review of 50 systematic reviews that used PROBAST to appraise 2104 prediction models demonstrated unclear or high ROB, in particular of the *Analysis* domain [50]. The latter results were markedly stable over time, highlighting the urgent need to consider ROB in prediction studies. Generally, systematic reviews of prediction models in other dental fields, such as for orthodontic treatment outcomes [51], for periodontitis [52], and for tooth loss and oral cancers [46] conclude that there is a lack of transparent reporting and identification of bias across included studies. As a consequence, predictive performance of the models is not possible to be fully assessed or compared quantitatively.

### Implications of the results for future practice and research

In this review, predictive performance was re-calculated and presented as LRs. In comparison with the commonly used sensitivity and specificity, LRs are considered to be more clinically meaningful [53, 54] as LRs have the advantage of incorporating all four cells of the 2 × 2 table, in contrast to sensitivity and specificity which makes use of only two cells. LRs +  ≥ 5.0 was selected as the threshold for prediction of caries increment, and this was achieved for only 5 models [17, 23, 26, 29]. To develop pertinent models, future investigations must address obvious deficiencies and avoid ROB in model design and investigation protocols. One key aspect is to verify the utility of predictors and the most useful set of predictors. In most included studies of the present review, predictors with a statistically significant association with the outcome were selected. As proposed in PROBAST, a better approach is to use non-statistical methods and select a few predictors based on existing knowledge in combination with reliability, consistency, applicability, availability, and costs of predictor measurement relevant to the targeted setting. Considering that numerous redundant predictors pose a burden in terms of availability and expenditure, it may be wise to reconsider the number of predictors included. Regarding studies in the current review, the performance of models with several predictors were inferior or equivalent to those of models based on fewer predictors, as demonstrated by Gao et al. [17].

The most prevalent predictor was c*aries experience*, expressed as a cavity, dentinal caries or filling in all but two studies. In adolescents, a considerable proportion of caries occurs as enamel lesions or as progression of enamel caries into dentinal caries [55, 56]. If the purpose of future prediction models is to take a preventive approach as regards the progression of lesions, it can be argued that the impact of prediction models will be limited if enamel lesions are not considered. Inclusion of enamel caries is also critical when evaluating and comparing results of interventions based on prediction models. Therefore, we recommend an implementation of a common language with criteria for dental caries also comprising enamel lesions, as described by ICDAS [34].

Another prevalent predictor was mutans streptococci in saliva included in all but 6 studies and in several networks with other predictors. The consistent inclusion of mutans streptococci can be attributed to that the studies of model development performed between 1992 and 2010 probably were influenced by the “Specific Plaque Hypothesis”, with mutans streptococci considered as the major etiological agent for caries [57]. By focusing on mutans streptococci, identified by growth on the selective MSB medium, the possibility to recognize other bacteria that exhibited an equally strong association with caries was disregarded in huge numbers of clinical studies. In a study using 16SDNA sequencing [58], it was demonstrated that more than 20 different colony forming units resembling the morphology of mutans streptococci colonies on MSB agar were in fact not mutans streptococci but identified as, e.g., *Streptococcus sanguinis* or *Streptococcus anginosus.* Caries does occur in the absence of mutans streptococci, and several other acid-producing and acid tolerant microbial species might contribute to caries development [59]. In other words, mutans streptococci in saliva might have been overestimated as a predictor, while the impact of other microbiota has been underestimated.

As suggested by Fontana et al. [3], new predictors, such as microbiota composition and metabolomics of dental plaque or saliva, should be considered in the future. As illustrated in a model of the caries process (Fig. 3), predictors at the societal structural, tooth, and physiological levels at the top of the model do not command causal associations with events close to demineralization of dental tissues. Unless predictors from the top levels carry over to predictors at the lower levels, such predictors will not improve the predictive performance. The necessary condition for demineralization is prolonged periods of low pH in dental plaque (below pH 5.5) (Fig. 3). The former will only occur if most of the dental plaque microbiota is acid tolerant. Therefore, we propose that attention should be given to a specific phenotype of bacteria (i.e., acid tolerant) as predictor instead of a specific genotype (e.g., mutans streptococci) as an additional predictor to caries experience. Our proposal is in line with the *“*Ecological Plaque Hypothesis” for caries [60, 61]. Frequent intake of fermentable carbohydrates resulting in lactic acid production is the driving force to create low pH conditions in dental plaque, provoking acid adaptation of bacteria that result in further enhanced acid production (Fig. 3). If the acidic conditions persist, the most adept acid tolerant bacteria will be selected and the mineral balance that accelerates demineralisation will be disturbed further. In this way, protons (H^+^) induced by saccharolytic bacteria in dental plaque, are responsible for both demineralization of dental tissues and acid adaptation of plaque bacteria. Future studies should be encouraged to verify the utility of biomarker predictors and the most useful predictor combinations, in line with the proposed caries process model.

## Conclusions

The results of model performance should be interpreted with caution due to shortcomings in the design, execution, and reporting of the included studies. The modest performance of most models leads us to question the inclusion of a wide range of predictors and to underline the importance of selecting a few predictors based on their applicability, availability, and costs. Hence, in an effort to identify non-redundant predictors, based on existing knowledge of the caries process, attention should be given to acid tolerant bacteria in the dental plaque. Our critical appraisal of the studies of caries prediction models highlighted methodological deficiencies and inadequate reporting. Shortcomings in study design, conduct and analysis can affect the predictive ability of the models. Flawed or distorted estimates will lead to uncertainty about the prediction. Nevertheless, the models are presented continuously in the dental scientific literature, utilized in dental education and applied in clinical decision-making.

### Supplementary Information

**Additional file 1.** PRISMA 2020 Checklist.

**Additional file 2.** MEDLINE search for study selection.

**Additional file 3.** Excluded full-text studies with reasons for exclusion.

**Additional file 4.** Detailed description of included studies as supplementary information to Table 1.

**Additional file 5.** Reference list of publications describing methodology for predictors presented in Additional file 4.

**Additional file 6.** Responses to signalling questions of PROBAST [6].

**Additional file 7.** Distribution of predictors by Level and category in multivariable developmental models of coronal caries increment. Predictors included in final models highlighted in red.

## Data Availability

In addition to data in Supplementary information presented in Additional files “Protocol for inclusion and exclusion of full text studies” and “Protocol for data extraction according to CHARMS” are available from the corresponding author in reasonable request.

## Abbreviations

AUC: Area under receiver operating curve
CAMBRA: Caries Management by Risk Assessment
CAT: Caries-risk Assessment Tool
CHARMS: The CHecklist for critical Appraisal and data extraction for systematic Reviews of prediction Modelling Studies
EPV: Number of events in relation to number of variables; ICDAS: International Caries Detection and Assessment System
LR +: Positive likelihood ratio
LR −: Negative likelihood ratio
MSB: Mitis-Salivarius Bacitracin medium
PROBAST: The Prediction model Risk Of Bias ASsessment Tool
ROB: Risk of bias

## References

[1] Collins GS, de Groot JA, Dutton S, Omar O, Shanyinde M, Tajar A (2014). External validation of multivariable prediction models: a systematic review of methodological conduct and reporting. BMC Med Res Methodol.

[2] Kassebaum NJ, Smith NJ, Bernabé E, Fleming TD, Reynolds AE, Vos T (2017). Global, regional, and national prevalence, incidence, and disability adjusted life years for oral conditions for 195 countries, 1990–2015: a systematic analysis for the global burden of diseases, injuries, and risk factors. J Dent Res.

[3] Fontana M, Carrasco-Labra A, Spallek H, Eckert G, Katz B (2020). Improving caries risk prediction modeling: a call for action. J Dent Res.

[4] Moons KGM, de Groot JAH, Bouwmeester W, Vergouwe Y, Mallett S, Altman DG, et al. Critical appraisal and data extraction for systematic reviews of prediction modelling studies: the CHARMS checklist. PLoS Med. 2014;11:e1001744. 10.1371/journal.pmed.1001744. 10.1371/journal.pmed.1001744 PMC4196729 25314315

[5] Wolff RF, Moons KGM, Riley RD, Whiting PF, Westwood M, Collins GS (2019). PROBAST: a tool to assess the risk of bias and applicability of prediction model studies. Ann Intern Med.

[6] Moons KGM, Wolff RF, Riley RD, Whiting PF, Westwood M, Collins GS (2019). PROBAST: a tool to assess risk of bias and applicability of prediction model studies: explanation and elaboration. Ann Intern Med.

[7] Page MJ, McKenzie JE, Bossuyt PM, Boutron I, Hoffmann TC, Mulrow CD, et al. The PRISMA 2020 statement: an updated guideline for reporting systematic reviews. BMJ. 2021;372:n71. 10.1136/bmj.n71. 10.1136/bmj.n71 PMC8005924 33782057

[8] Slade GD, Caplan DJ (2000). Impact of analytic conventions on outcome measures in two longitudinal studies of dental caries. Community Dent Oral Epidemiol.

[9] Tellez M, Gomez J, Pretty I, Ellwood R, Ismail AI (2013). Evidence on existing caries risk assessment systems: are they predictive of future caries?. Community Dent Oral Epidemiol.

[10] Mejàre I, Axelsson S, Dahlén G, Espelid I, Norlund A, Tranæus S (2014). Caries risk assessment. A systematic review Acta Odontol Scand.

[11] Cagetti MG, Bontà G, Cocco F, Lingstrom P, Strohmenger L, Campus G (2018). Are standardized caries risk assessment models effective in assessing actual caries status and future caries increment?. A systematic review BMC Oral Health.

[12] Jørgensen MR, Twetman S (2020). A systematic review of risk assessment tools for early childhood caries: is there evidence?. Eur Arch Paediatr Dent.

[13] Angulo M, Zinemanas E, Pivel L, Jorysz E, Casamayou R, Krasse B (1995). Caries incidence, effect of preventive measures, and caries prediction in Uruguayan children. Acta Odontol Scand.

[14] Demers M, Brodeur JM, Mouton C, Simard PL, Trahan L, Veilleux G (1992). A multivariate model to predict caries increment in Montreal children aged 5 years. Community Dent Health.

[15] Disney JA, Graves RC, Stamm JW, Bohannan HM, Abernathy JR, Zack DD (1992). The University of North Carolina caries risk assessment study: further developments in caries risk prediction. Community Dent Oral Epidemiol.

[16] Fontana M, Santiago E, Eckert GJ, Ferreira-Zandona AG (2011). Risk factors of caries progression in a Hispanic school-aged population. J Dent Res.

[17] Gao XL, Hsu CY, Xu Y, Hwarng HB, Loh T, Koh D (2010). Building caries risk assessment models for children. J Dent Res.

[18] Hänsel Petersson G, Twetman S, Bratthall D (2002). Evaluation of a computer program for caries risk assessment in schoolchildren. Caries Res.

[19] Pang L, Wang K, Tao Y, Zhi Q, Zhang J, Lin H. A new model for caries risk prediction in teenagers using a machine learning algorithm based on environmental and genetic factors.Front Genet. 2021;12:636867. 10.3389/fgene.2021.636867. 10.3389/fgene.2021.636867 PMC7990890 33777105

[20] Sánchez-Pérez L, Golubov J, Irigoyen-Camacho ME, Moctezuma PA, Acosta-Gio E (2009). Clinical, salivary, and bacterial markers for caries risk assessment in schoolchildren: a 4-year follow-up. Int J Paediatr Dent.

[21] Powell LV, Mancl LA, Senft GD (1991). Exploration of prediction models for caries risk assessment of the geriatric population. Community Dent Oral Epidemiol.

[22] Ritter AV, Preisser JS, Puranik CP, Chung Y, Bader JD, Shugars DA (2016). A predictive model for root caries incidence. Caries Res.

[23] Sánchez-García S, Reyes-Morales H, Juárez-Cedillo T, Espinel-Bermúdez C, Solórzano-Santos F, García-Peña C (2011). A prediction model for root caries in an elderly population. Community Dent Oral Epidemiol.

[24] Beck JD, Weintraub JA, Disney JA, Graves RC, Stamm JW, Kaste LM (1992). University of North Carolina caries risk assessment study: comparisons of high risk prediction, any risk prediction, and any risk etiologic models. Community Dent Oral Epidemiol.

[25] Birpou E, Agouropoulos A, Twetman S, Kavvadia K (2019). Validation of different Cariogram settings and factor combinations in preschool children from areas with high caries risk. Int J Paediatr Dent.

[26] Campus G, Cagetti MG, Sale S, Carta G, Lingström P (2012). Cariogram validity in schoolchildren: a two-year follow-up study. Caries Res.

[27] Christian B, Calache H, Adams G, Hall M, Dashper S, Gibbs L, et al. A methodological study to assess the measurement properties (reliability and validity) of a caries risk assessment tool for young children. J Dent. 2020;95:103324. 10.1016/j.jdent.2020.103324. 10.1016/j.jdent.2020.103324 32200008

[28] Dolic O, Obradovic M, Kojic Z, Trtic N, Sukara S, Knezevic N (2020). Validation of Cariogram in caries prediction in women and their children 4 years after pregnancy – longitudinal study. Risk Manag Healthc Policy.

[29] Gao X, Di Wu I, Lo EC, Chu CH, Hsu CY, Wong MC (2013). Validity of caries risk assessment programmes in preschool children. J Dent.

[30] Petersson GH, Twetman S (2015). Caries risk assessment in young adults: a 3 year validation of the Cariogram model. BMC Oral Health.

[31] Petersson GH, Isberg PE, Twetman S (2010). Caries risk profiles in schoolchildren over 2 years assessed by Cariogram. Int J Paediatr Dent.

[32] Lif Holgerson P, Twetman S, Stecksén-Blicks C (2009). Validation of an age-modified caries risk assessment program (Cariogram) in preschool children. Acta Odontol Scand.

[33] Hayes M, Da Mata C, McKenna G, Burke FM, Allen PF (2017). Evaluation of the Cariogram for root caries prediction. J Dent.

[34] Ismail AI, Sohn W, Tellez M, Amaya A, Sen A, Hasson H (2007). The International Caries Detection and Assessment System (ICDAS): an integrated system for measuring dental caries. Community Dent Oral Epidemiol.

[35] Pitts NB, Ekstrand KR, ICDAS Foundation. International Caries Detection and Assessment System (ICDAS) and its International Caries Classification and Management System (ICCMS) – methods for staging of the caries process and enabling dentists to manage caries. Community Dent Oral Epidemiol. 2013;41:e41–52. 10.1111/cdoe.12025. 10.1111/cdoe.12025 24916677

[36] Aldin A, Umlauff L, Estcourt LJ, Collins G, Moons KG, Engert A, et al. Interim PET-results for prognosis in adults with Hodgkin lymphoma: a systematic review and meta-analysis of prognostic factor studies. Cochrane Database Syst Rev. 2020;1:CD012643. 10.1002/14651858.CD012643.pub3. 10.1002/14651858.CD012643.pub3 PMC6984446 31930780

[37] Deeks JJ (2001). Systematic reviews in health care: systematic reviews of evaluations of diagnostic and screening tests. BMJ.

[38] Koopman PAR (1984). Confidence intervals for the ratio of two binomial proportions. Biometrics.

[39] Oral health surveys — basic methods. 2nd ed. Geneva: World Health Organization, 1979.

[40] WHO, World Health Organization. Oral health surveys: basic methods. 3rd ed. Geneva: WHO, 1987.

[41] World Health Organization. Oral health surveys: basic methods, 4th ed. Geneva: World Health Organization, 1997. https://apps.who.int/iris/handle/10665/41905.

[42] Radike AW. Criteria for diagnosis of dental caries. Proceeding of the conference on the clinical testing of cariostatic agents, 1968. Chicago: Am Dent Assoc. 1972. pp 92–5.

[43] Pitts NB, Longbottom C (1995). Preventive care advised (PCA)/operative care advised (OCA) – categorising caries by the management option. Community Dent Oral Epidemiol.

[44] Struyf T, Deeks JJ, Dinnes J, Takwoingi Y, Davenport C, Leeflang MM, et al. Signs and symptoms to determine if a patient presenting in primary care or hospital outpatient settings has COVID-19 disease. Cochrane Database Syst Rev. 2020;7:CD013665. 10.1002/14651858.CD013665. 10.1002/14651858.CD013665 PMC7386785 32633856

[45] Moons KG, Altman DG, Reitsma JB, Ioannidis JP, Macaskill P, Steyerberg EW (2015). Transparent Reporting of a multivariable prediction model for Individual Prognosis or Diagnosis (TRIPOD): explanation and elaboration. Ann Intern Med.

[46] Du M, Haag D, Song Y, Lynch J, Mittinty M (2020). Examining bias and reporting in oral health prediction modeling studies. J Dent Res.

[47] Lasserson TJ, Thomas J, Higgins JPT, Higgins JPT, Thomas J, Chandler J, Cumpston M, Li T, Page MJ, Welch VA (2019). Chapter 1: Starting a review. Cochrane Handbook for Systematic Reviews of Interventions.

[48] Hintze H, Wenzel A, Danielsen B, Nyvad B (1998). Reliability of visual examination, fibre-optic transillumination, and bite-wing radiography, and reproducibility of direct visual examination following tooth separation for the identification of cavitated carious lesions in contacting approximal surfaces. Caries Res.

[49] Su N, Lagerweij MD, van der Heijden GJMG. Assessment of predictive performance of caries risk assessment models based on a systematic review and meta-analysis. J Dent. 2021;110:103664. 10.1016/j.jdent.2021.103664. 10.1016/j.jdent.2021.103664 33984413

[50] de Jong Y, Ramspek CL, Zoccali C, Jager KJ, Dekker FW, van Diepen M (2021). Appraising prediction research: a guide and meta-review on bias and applicability assessment using the Prediction model Risk Of Bias ASsessment Tool (PROBAST). Nephrology.

[51] Fudalej P, Dragan M, Wedrychowska-Szulc B (2011). Prediction of the outcome of orthodontic treatment of Class III malocclusions-a systematic review. Eur J Orthod.

[52] Du M, Bo T, Kapellas K, Peres MA (2018). Prediction models for the incidence and progression of periodontitis: a systematic review. J Clin Periodontol.

[53] Chien PF, Khan KS. Evaluation of a clinical test. II: assessment of validity. BJOG.2001;108:568–72. 10.1111/j.1471-0528.2001.00128.x. 10.1111/j.1471-0528.2001.00128.x 11426889

[54] Grimes DA, Schulz KF (2005). Refining clinical diagnosis with likelihood ratios. Lancet.

[55] Lith A, Lindstrand C, Gröndahl H-G. Caries development in a young population managed by a restrictive attitude to radiography and operative intervention: II. A study at the surface level. Dentomaxillofac Radiol. 2002;31:232–9. 10.1038/sj.dmfr.4600705. 10.1038/sj.dmfr.4600705 12087440

[56] Mejàre I, Stenlund H, Zelezny-Holmlund C. Caries incidence and lesion progression from adolescence to young adulthood: a prospective 15-year cohort study in Sweden. Caries Res. 2004;38:130-41. 10.1159/000075937. 10.1159/000075937 14767170

[57] Loesche WJ (1979). Clinical and microbiological aspects of chemotherapheutic agents used according the specific plaque hypothesis. J Dent Res.

[58] Yoo SY, Kim PS, Hwang HK, Lim S-H, Kim K-W, Choe S-J (2005). Identification of non-mutans streptococci organisms in dental plaques recovering on mitis-salivarius bacitracin agar medium. J Microbiol.

[59] Beighton D (2005). The complex oral microflora of high-risk individuals and groups and its role in the caries process. Community Dent Oral Epidemiol.

[60] Marsh PD (2003). Are dental diseases examples of ecological catastrophes?. Microbiology (Reading).

[61] Marsh PD, Takahashi N, Nyvad B. Biofilms in caries development. In: Fejerskov O, Nyvad B, Kidds E, editors. Dental caries. The disease and its clinical management. Oxford: Wiley Blackwell; 2015. p.108–31.

