# Supplementary material for: Multivariable prediction models of caries increment: a systematic review and critical appraisal
Source: Syst Rev. 2023 Oct 30;12:202. doi: 10.1186/s13643-023-02298-y (PMC10614348; doi:10.1186/s13643-023-02298-y)
Supplement: Supplementary file 2 — Additional file 2. MEDLINE search for study selection. [file 13643_2023_2298_MOESM2_ESM.pdf]

Additional file 2. MEDLINE search for records via PubMed April 23, 2021

((((((((((((((((((((("ROC Curve"[MeSH]) OR "Follow-Up Studies"[MeSH]) OR ("Sensitivity and Specificity"[Mesh])) OR "Area under Curve"[MeSH]) OR "Odds Ratio"[MeSH]) OR prospective[Title/Abstract]) OR sensitivity[Title/Abstract]) OR specificity[Title/Abstract]) OR "follow up"[Title/Abstract]) OR evaluat*[Title/Abstract]) OR odds ratio*[Title/Abstract]) OR ROC curve*[Title/Abstract]) OR ROC analys*[Title/Abstract]) OR receiver operating characteristic*[Title/Abstract]) OR area under curve*[Title/Abstract]) OR AUC[Title/Abstract]) OR outcome*[Title/Abstract]) OR validat*[Title/Abstract])) AND ((((((("Dental Caries"[Mesh]) OR carious dentin*[Title/Abstract]) OR dental caries*[Title/Abstract]) OR dental decay*[Title/Abstract]) OR dental fissure*[Title/Abstract]) OR root caries*[Title/Abstract]) OR cervical caries*[Title/Abstract])) AND ((((((((((((((((("Risk Assessment"[Mesh]) OR risk assessment*[Title/Abstract]) OR risk calculat*[Title/Abstract]) OR risk model*[Title/Abstract]) OR risk prediction*[Title/Abstract]) OR risk profile*[Title/Abstract]) OR decision support tool*[Title/Abstract]) OR clinical decision support system*[Title/Abstract]) OR predictive model*[Title/Abstract]) OR predictive equation*[Title/Abstract]) OR statistical model*[Title/Abstract]) OR prognostic model*[Title/Abstract]) OR CAMBRA[Title/Abstract]) OR CAT[Title/Abstract]) OR "Dundee Caries Risk Assessment Model"[Title/Abstract]) OR NUS-CRA[Title/Abstract]) OR previser[Title/Abstract]))) NOT (((((("News"[pt]) OR "Bibliography"[pt]) OR "Letter"[pt]) OR "Editorial"[pt]) OR "Comment"[pt]) OR "Case Reports"[pt]). Filter: English

A combination of specified medical subject headings (MeSH) terms and keywords was drafted in collaboration with librarians and peer-reviewed to ensure comprehensiveness. The search strategy was pre-tested in the MEDLINE database and subsequently adapted to the syntax and subject headings of the other databases.

The final search was developed for use in MEDLINE via PubMed adapting the search to other databases including Cochrane via Wiley and Web of Science on April 23, 2021.
