# Supplementary material for: Multivariable prediction models of caries increment: a systematic review and critical appraisal
Source: Syst Rev. 2023 Oct 30;12:202. doi: 10.1186/s13643-023-02298-y (PMC10614348; doi:10.1186/s13643-023-02298-y)
Supplement: Supplementary file 3 — Additional file 3. Excluded full-text studies with reasons for exclusion. [file 13643_2023_2298_MOESM3_ESM.pdf]

**Additional file 3.** List of excluded full-text studies with reasons for exclusion.

*Reason for exclusion*
1 Outcome: Not caries increment at individual level
2 Design: Not longitudinal study (no prognostic prediction)
3 Outcome: <3 variables/predictors in final model
4 Timing: Follow-up time <1 year
5 Intervention: Description of predictors or outcome unclear
6 Outcome: Model performance not or incorrectly presented or only area under curve (AUC) is presented
7 Duplicate study of the same model and/or sample
8 Setting: Deviant

| **Study** | **Reason** |
|---|---|
| Abanto J, Celiberti P, Braga MM, Vidigal EA, Cordeschi T, Haddad AE, et al. Effectiveness of a preventive program based on caries risk assessment and recall intervals on the incidence and regression of initial caries lesions in children. Int J Paediatr Dent. 2015;2:291-9. | 4 |
| Abernathy JR, Graves RC, Bohannan HM, Stamm JW, Greenberg BG, Disney JA. Development and application of a prediction model for dental caries. Community Dent Oral Epidemiol. 1987;15:24-8. | 5 |
| Agouropoulos A, Birpou E, Twetman T, Kavvadia K. Validation of three caries risk assessment tools for preschool children from areas with high caries prevalence. Pediatr Dent. 2019;41:391-9. | 6 |
| Al Mulla AH, Kharsa SA, Kjellberg H, Birkhed D. Caries risk profiles in orthodontic patients at follow-up using Cariogram. Angle Orthod. 2009;79:323-30. | 6 |
| Alaluusua S, Kleemola-Kujala E, Grönroos L, Evälahti M. Salivary caries-related tests as predictors of future caries increment in teenagers. A three-year longitudinal study. Oral Microbiol Immunol. 1990;5:77-8. | 3 |
| Alaluusua S. Salivary counts of mutans streptococci and lactobacilli and past caries experience in caries prediction. Caries Res. 1993;27 Suppl 1:68-71. | 3 |
| Arino M, Ito A, Fujiki S, Sugiyama S, Hayashi M. Multicenter study on caries risk assessment in adults using survival classification and regression trees. Adv in Dent Res. 2018;29:15-23. | 3 |
| Axelsson P, Paulander J, Svärdström G, Tollskog G, Nordensten S. Integrated caries prevention: effect of a needs-related preventive program on dental caries in children. County of Värmland, Sweden: results after 12 years. Caries Res. 1993;27 Suppl 1:83-94. | 4 |
| Azrak B, Gleissner C, Willershausen B, Jadamus-Stöcker J, Callaway A. Accuracy of a chair-side test for predicting caries risk compared with established methods. A pilot study. Schweiz Monatsschr Zahnmed. 2010;120:409-14. | 3 |
| Baca P, Parejo E, Bravo M, Castillo A, Liébana J. Discriminant ability for caries risk of modified colorimetric tests. Med Oral Patol Oral Cir Bucal. 2011;16:e978-83. | 3 |
| Bader JD, Graves RC, Disney JA, Bohannan HM, Stamm JW, Abernathy JR, et al. Identifying children who will experience high caries increments. Comnunity Dent Oral Epidemiol. 1986;14:198-201. | 5 |
| Bader JD, Perrin NA, Maupomé G, Rush WA, Rindal BD. Exploring the contributions of components of caries risk assessment guidelines. Community Dent Oral Epidemiol. 2008;36:357-62. | 3 |
| Bartoloni JA, Chao SY, Martin GC, Caron GA. Dental caries risk in the U.S. Air Force. J Am Dent Assoc. 2006;137:1582-91. | 1 |
| Beck JD, Lawrence HP, Koch GG. A method for adjusting caries increments for reversals due to examiner misclassification. Community Dent Oral Epidemiol. 1995;23: 321-30. | 1 |
| Bernabé E, MacRitchie H, Longbottom C, Pitts NB, Sabbah W. Birth weight, breastfeeding, maternal smoking and caries trajectories. J Dent Res. 2017;96:171-8. | 3 |

| | |
|---|---|
| Bernabé E, Vehkalahti MM, Sheiham A, Aromaa A, Suominen AL. Sugar-sweetened beverages and dental caries in adults: a 4-year prospective study. J Dent. 2014;42:952-8. | 3 |
| Birkeland JM, Brock L, Jorkjend L. Caries experience as predictor for caries incidence. Community Dent Oral Epidemiol. 1976;4:66-9. | 6 |
| Bjarnason S, Köhler B. Caries risk assessment in adolescents. Swed Dent J. 1997;21:41-8. | 3 |
| Borghi GN, Rodrigues LP, Lopes LM, Parisotto TM, Steiner-Oliveira C, Nobre-Dos-Santos M. Relationship among α amylase and carbonic anhydrase VI in saliva, visible biofilm, and early childhood caries: a longitudinal study. Int J Paediatr Dent. 2017;2:174-82. | 3 |
| Brons-Piche E, Eckert GJ, Fontana M. Predictive validity of a caries risk assessment model at a dental school. J Dent Educ. 2019; 83:144-50. | 6 |
| Busby M, Chapple E, Matthews R, Chapple IL. Practitioner evaluation of a novel online integrated oral health and risk assessment tool: a practice pilot. Br Dent J. 2013;215:115-20. | 2 |
| Celik EU, Gokay N, Ates M. Efficiency of caries risk assessment in young adults using Cariogram. Eur J Dent. 2012;6:270-9. | 6 |
| Chang J, Kim HY. Does caries risk assessment predict the incidence of caries for special needs patients requiring general anesthesia? Acta Odontol Scand. 2014;72:721-8. | 4 |
| Chang J, Lee JH, Son HH, Kim HY. Caries risk profile of Korean dental patients with severe intellectual disabilities. Spec Care Dentist. 2014;34:201-7. | 2 |
| Chankanka O, Cavanaugh JE, Levy SM, Marshall TA, Warren JJ, Broffitt B, et al. Longitudinal associations between children's dental caries and risk factors. J Public Health Dent. 2011;71:289-300. | 3 |
| Chaussain C, Opsahl Vital S, Viallon V, Vermelin L, Haignere C, Sixou M, et al. Interest in a new test for caries risk in adolescents undergoing orthodontic treatment. Clin Oral Investig. 2010;14:177-85. | 6 |
| Cheng J, Chaffee BW, Cheng NF, Gansky SA, Featherstone JD. Understanding treatment effect mechanisms of the CAMBRA randomized trial in reducing caries increment. J Dent Res. 2015;94:44-51. | 2 |
| David J, Raadal M, Wang NJ, Strand GV. Caries increment and prediction from 12 to 18 years of age: a follow-up study. Eur Arch Paediatr Dent. 2006;7:31-7. | 3 |
| De Marchi RJ, Dos Santos CM, Martins AB, Hugo FN, Hilgert JB, Padilha DM. Four-year incidence and predictors of coronal caries in south Brazilian elderly. Community Dent Oral Epidemiol. 2015;43:452-60. | 3 |
| Disney JA, Abernathy JR, Graves RC, Mauriello SM, Bohannan HM, Zack DD. Comparative effectiveness of visual/tactile and simplified screening examinations in caries risk assessment. Community Dent Oral Epidemiol. 1992;20:326-32. | 1 |
| Disney JA, Graves RC, Stamm JW, Bohannan HM, Abernathy JR. The University of North Carolina caries risk assessment study. II. Baseline caries prevalence. J Public Health Dent 1990;50:178-85. | 1 |
| Divaris K, Fisher EL, Shugars DA. White RP Jr. Risk factors for third molar occlusal caries: a longitudinal clinical investigation. J Oral Maxillofac Surg. 2012;70:1771-80. | 1 |
| Doméjean S, White JM, Featherstone JD. Validation of the CDA CAMBRA caries risk assessment--a six-year retrospective study. J Calif Dent Assoc. 2011;39:709-15. | 3 |
| Dou L, Luo J, Fu X, Tang Y, Gao J, Yang D. The validity of caries risk assessment in young adults with past caries experience using a screening Cariogram model without saliva tests. Int Dent J. 2018;68:221-6. | 6 |
| Drake CW, Beck JD, Lawrence HP, Koch GG. Three-year coronal caries incidence and risk factors in North Carolina elderly. Caries Res. 1997;31:1-7. | 5 |
| Ekstrand KR, Bruun G, Bruun M. Plaque and gingival status as indicators for caries progression on approximal surfaces. Caries Res 1998;32:41-5. | 3 |
| Enerbäck H, Lingström P, Möller M, Nylén C, Bresin CÖ, Ros IÖ, et al. Validation of caries risk assessment methods in orthodontic patients. Am J Orthod Dentofacial Orthop. 2020;158:92-101.e3. | 6 |

| | |
|---|---|
| Featherstone JD, White JM, Hoover CI, Rapozo-Hilo M, Weintraub JA, Wilson RS, et al. A randomized clinical trial of anticaries therapies targeted according to risk assessment (caries management by risk assessment). Caries Res. 2012;46:118-29. | 6 |
| Fontana M, Eckert GJ, Keels MA, Jackson R, Katz B, Levy BT, et al. Fluoride use in health care settings: association with children's caries risk. Adv Dent Res. 2018;29:24-34. | 1 |
| Gao XL, Lo EC, Chu CH, Hsu SC. Caries risk assessment programmes for Hong Kong children. Hong Kong Med J. 2015;21 Suppl 6:42-6. | 7 |
| Garg A, Madan M, Dua P, Saini S, Mangla R, Singhal P, et al. Validating the usage of Cariogram in 5- and 12-year-old school-going children in Paonta Sahib, Himachal Pradesh, India: a 12-month prospective study. Int J Clin Pediatr Dent. 2018;11:110-5. | 5 |
| Gauba K, Goyal A, Mittal N. A CAMBRA model for high caries risk Indian children: a pragmatic comprehensive tailored intervention. J Clin Pediatr Dent. 2016;40:36-43. | 1 |
| Gilbert GH, Duncan RP, Dolan TA, Foerster U. Twenty-four-month incidence of root caries among a diverse group of adults. Caries Res. 2001;35:366-75. | 3 |
| Graves RC, Abernathy JR, Disney JA, Stamm JW, Bohannan HMJ. University of North Carolina caries risk assessment study. III. Multiple factors in caries prevalence. Public Health Dent. 1991;51:134-43. | 1 |
| Grier A, Myers JA, O'Connor TG, Quivey RG, Gill SR, Kopycka-Kedzierawski DT. Oral microbiota composition predicts early childhood caries onset. J Dent Res. 2021;100:599-607. | 3 |
| Grindefjord M, Dahllöf G, Nilsson B, Modéer T. Stepwise prediction of dental caries in children up to 3.5 years of age. Caries Res. 1996;30:256-66. | 5 |
| Grindefjord M, Dahllöf G, Modéer T. Caries development in children from 2.5 to 3.5 years of age: a longitudinal study. Caries Res. 1995;29:449-54. | 3 |
| Guedes RS, Piovesan C, Ardenghi TM, Emmanuelli B, Braga MM, Ekstrand KR, et al. Validation of visual caries activity assessment: a 2-yr cohort study. J Dent Res. 2014;93:101S-107S. | 3 |
| Ha DH, Spencer AJ, Slade GD, Chartier AD. The accuracy of caries risk assessment in children attending South Australian School Dental Service: a longitudinal study. BMJ Open. 2014;4:e004311. | 4 |
| Hallett KB, O'Rourke PK. Baseline dental plaque activity, mutans streptococci culture, and future caries experience in children. Pediatr Dent. 2013;35:523-8. | 1 |
| Hallett KB. The application of caries risk assessment in minimum intervention dentistry. Aust Dent J. 2013;58:Suppl 1:26-34. | 1 |
| Hart TC, Corby PM, Hauskrecht M, Hee Ryu O, Pelikan R, Valko M, et al. Identification of microbial and proteomic biomarkers in early childhood caries. Int J Dent. 2011;2011:196721. | 2 |
| Hawkins RJ, Jutai DK, Brothwell DJ, Locker D. Three-year coronal caries incidence in older Canadian adults. Caries Res. 1997;31:405-10. | 6 |
| Helderman WH, Mulder J, van'T Hof MA, Truin GJ. Validation of a Swiss method of caries prediction in Dutch children. Community Dent Oral Epidemiol. 2001;29:341-5. | 3 |
| Holst A, Braune K. Dental assistants' ability to select caries risk-children and to prevent caries. Swed Dent J. 1994;18:243-9. | 6 |
| Holst A, Mårtensson I, Laurin M. Identification of caries risk children and prevention of caries in pre-school children. Swed Dent J. 1997;21:185-91. | 7 |
| Hänsel Petersson G, Fure S, Bratthall D. Evaluation of a computer-based caries risk assessment program in an elderly group of individuals. Acta Odontol Scand. 2003;61:164-71. | 6 |
| Ismail AI, Sohn W, Lim S, Willem JM. Predictors of dental caries progression in primary teeth. J Dent Res. 2009;88:270-5. | 3 |
| Ito A, Hayashi M, Hamasaki T, Ebisu S. How regular visits and preventive programs affect onset of adult caries. J Dent Res. 2012;91(7 Suppl):52S-58S. | 3 |

| | |
|---|---|
| Ito, A, Hayashi, M, Hamasaki, T. Ebisu, S. Risk assessment of dental caries by using classification and regression trees. J Dent. 2011;39:457-63. | 3 |
| Kalhan TA, Un Lam C, Karunakaran B, Chay PL, Chng CK, Nair R, et al. Caries risk prediction models in a medical health care setting. J Dent Res. 2020;99:787-96. | 8 |
| Kalwitzki M, Weiger R, Axmann-Krcmar D, Rosendahl R. Caries risk analysis: considering caries as an individual time-dependent process. Int J Paediatr Dent. 2002;12:132-42. | 3 |
| Kemparaj U, Chavan S, Shetty NL. Caries risk assessment among school children in Davangere city using Cariogram. Int J Prev Med. 2014;5:664-71. | 6 |
| Kim JM, Choi JS, Choi YH, Kim HE. Simplified prediction model for accurate assessment of dental caries risk among participants aged 10-18 years. Tohoku J Exp Med. 2018;246:81-6. | 2 |
| Kingman A, Little W, Gomez I, Heifetz SB, Driscoll WS, Sheats R, et al. Salivary levels of Streptococcus mutans and lactobacilli and dental caries experiences in a US adolescent population. Community Dent Oral Epidemiol. 1988;16:98-103. | 3 |
| Klock B, Krasse B. A comparison between different methods for prediction of caries activity. Scand J Dent Res. 1979;87:129-39. | 5 |
| Kopycka-Kedzierawski DT, Billings RJ. Application of nonhomogeneous Markov models for analyzing longitudinal caries risk. Community Dent Oral Epidemiol. 2006;34:123-9. | 3 |
| Kuru E, Eden E. Success of two caries risk assessment tools in children: a pilot study with a 3-year follow-up. Int Q Community Health Educ. 2020;40:317-20. | 5 |
| Lawrence HP, Hunt RJ, Beck JD. Three-year root caries incidence and risk modeling in older adults in North Carolina. J Public Health Dent. 1995;55:69-78. | 3 |
| Lee HJ, Kim JB, Jin BH, Paik DI, Bae KH. Risk factors for dental caries in childhood: a five-year survival analysis. Community Dent Oral Epidemiol. 2015;43:163-71. | 3 |
| Leroy R, Declerck D. Impact of caries onset on number and distribution of new lesions in preschool children. Int J Paediatr Dent. 2013;23:39-47. | 1 |
| Litt MD, Reisine S, Tianoff N. Multidimensional causal model of dental caries development in low-income preschool children. Public Health Rep. 1995;110:607-17. | 6 |
| MacRitchie HMB, Longbottom C, Robertson M, Nugent Z, Chan K, Radford JR, et al. Development of the Dundee Caries Risk Assessment Model (DCRAM) --risk model development using a novel application of CHAID analysis. Community Dent Oral Epidemiol. 2012;40:37-45. | 5 |
| Maserejian NN, Tavares MA, Hayes C, Soncini JA, Trachtenberg FL. Prospective study of 5-year caries increment among children receiving comprehensive dental care in the New England children's amalgam trial. Community Dent Oral Epidemiol. 2009;37:9-18. | 6 |
| Mattiasson-Robertsson A, Twetman S. Prediction of caries incidence in schoolchildren living in a high and a low fluoride area. Community Dent Oral Epidemiol. 1993;21:365-9. | 3 |
| Morou-Bermudez E, Elias-Boneta A, Billings RJ, Burne RA, Garcia-Rivas V, Brignoni-Nazario V, et al. Urease activity as a risk factor for caries development in children during a three-year study period: a survival analysis approach. Arch Oral Biol. 2011;56:1560-8. | 3 |
| Mortazavi S, Enshaei Z, Farajzadegan Z. Development of caries risk assessment tool for Iranian preschoolers: a primary validation study. Int J Prev Med. 2017;8:92 | 2 |
| Nemeth L, Groselj M, Golez A, Arhar A, Frangez I, Cankar K. The impact of photobiomodulation of major salivary glands on caries risk. Lasers Med Sci. 2020;35:193-203. | 4 |
| Nishi M, Kelleher V, Cronin M, Allen F. The effect of mobile personalised texting versus non-personalised texting on the caries risk of underprivileged adults: a randomised control trial. BMC Oral Health. 2019;19:44. | 4 |
| Nobre MA, Sezinando A, Fernandes I, Maló P. Risk score top predict dental caries in adult patients for use in the clinical setting. J Clin Med. 2019; 8:203. | 6 |

| | |
|---|---|
| Rechmann P, Chaffee BW, Rechmann BMT, Featherstone JDB. Changes in caries risk in a practice-based randomized controlled trial. Adv Dent Res. 2018;29:15-23. | 1 |
| Pearce EIF, Dong Y-M, Yue L, Gao X-J, Purdie GL, Wang J-D. Plaque minerals in the prediction of caries activity. Community Dent Oral Epidemiol. 2002;30:61-9. | 3 |
| Peltzer K, Mongkolchati A, Satchaiyan G, Rajchagool S, Pimpak T.  Sociobehavioral factors associated with caries increment: a longitudinal study from 24 to 36 months old children in Thailand. Int J Environ Res Public Health. 2014;11:10838-50. | 6 |
| Pereira da Silva Tagliaferro E, Pereira AC, Meneghim MC, Ambrosano GMB. Assessment of dental caries predictors in a seven-year longitudinal study. J Public Health Dent. 2006;66:169-79. | 3 |
| Peres MA, Barros AJ, Peres KG, Araújo CL, Menezes AM. Life course dental caries determinants and predictors in children aged 12 years: a population-based birth cohort. Community Dent Oral Epidemiol. 2009;37:123-33. | 3 |
| Petersson GH, Ericson E, Isberg PE, Twetman S. Caries risk assessment in young adults using public dental service guidelines and the Cariogram - a comparative study. Acta Odontol Scand. 2013;71:534-40. | 3 |
| Petersson GH, Fure S, Twetman S, Bratthall D. Comparing caries risk factors and risk profiles between children and elderly. Swed Dent J. 2004;28:119-28. | 1 |
| Petersson GH, Isberg PE, Twetman S. Caries risk profiles in schoolchildren over 2 years assessed by the Cariogram. Int J Paediatr Dent. 2010;20:341-6. | 7 |
| Pienihäkkinen K, Jokela J, Alanen P. Assessment of caries risk in preschool children. Caries Res. 2004;38:156-62. | 6 |
| Raitio M, Pienihäkkinen K, Scheinin A. Multifactorial modeling for prediction of caries increment in adolescents. Acta Odontol Scand. 1996;54:118-21. | 4 |
| Ravald N, Hamp SE. Prediction of root surface caries in patients treated for advanced periodontal disease. J Clin Periodontol. 1981;8:400-14. | 6 |
| Russell JI, MacFarlane TW, Aitchison TC, Stephen KW, Burchell CK. Prediction of caries increment in Scottish adolescents. Community Dent Oral Epidemiol. 1991;19:74-7. | 5 |
| Sánchez-Pérez L, Acosta-Gío E, Méndez-Ramírez I. A cluster analysis model for caries risk assessment. Arch Oral Biol. 2004;49:719-25. | 3 |
| Sánchez-Pérez L, Acosta-Gío E. Caries risk assessment from dental plaque and salivary Streptococcus mutans counts on two culture media. Arch Oral Biol. 2001;46:49-55. | 3 |
| Sarmadi R, Gabre P, Gahnberg L. Strategies for caries risk assessment in children and adolescents at public dental clinics in a Swedish county. Int J Paedatr Dent. 2009;19:135-40. | 1 |
| Scheinin A, Pienihäkkinen K, Tiekso J, Holmberg S. Multifactorial modeling for root caries prediction. Community Dent Oral Epidemiol. 1992;20:35-7. | 3 |
| Scheinin A, Pienihäkkinen K, Tiekso J, Holmberg S. Multifactorial modeling for root caries prediction: 3-year follow-up results. Community Dent Oral Epidemiol. 1994;22:126-9. | 3 |
| Schroeder U, Widenheim J, Peyron M, Hagg E. Prediction of caries in 1.5-year old children. Swed Dent J. 1994;18:95-104. | 5 |
| Seppä L, Hausen H. Frequency of initial caries lesions as predictor of future caries increment in children. Scand J Dent Res. 1998;96:9-13. | 3 |
| Skeie MS, Raadal M, Strand GV, Espelid I. The relationship between caries in the primary dentition at 5 years of age and permanent dentition. Int J Paediatr Dent. 2006;16:152-60. | 3 |
| Stamm JW, Disney A, Graves RC, Bohannan HM, Abernathy JR. The University of North Carolina caries risk assessment study I: rationale and content. J Public Health Dent. 1988; 48:225-32. | 5 |
| Stecksen-Blicks C, Holgerson PL, Twetman S. Caries risk profiles in two-year-old children from northern Sweden. Oral Health Prev Dent 2007;5:215-21. | 1 |

| | |
|---|---|
| Stewart PW, Stamm JW. Classification tree prediction models for dental caries from clinical, microbiological, and interview data. J Dent Res. 1991;70:1239-51. | 7 |
| Sudhir KM, Kanupuru KK, Fareed N, Mahesh P, Vandana K, Chaitra NT. CAMBRA as a tool for caries risk prediction among 12- to 13-year-old institutionalised children - a longitudinal follow-up study. Oral Health Prev Dent. 2016;14:355-62. | 6 |
| Takano N, Ando Y, Yoshihara A, Miyazaki H. Factors associated with root caries incidence in an elderly population. Community Dent Health. 2003;20: 217-22. | 3 |
| Tamaki Y, Nomura Y, Katsumura S, Okada A, Yamada H, Tsuge S, et al. Construction of a dental caries prediction model by data mining. J Oral Sci. 2009;51:61-8. | 1 |
| Un Lam C, Khin LW, Kalhan AC, Yee R, Lee YS, Chong MF, et al. Identification of caries risk determinants in toddlers: results of the GUSTO birth cohort study. Caries Res. 2017;51:271-82. | 2 |
| van Palenstein Helderman WH, van't Hof MA, van Loveren C. Prognosis of caries increment with past caries experience variables. Caries Res. 2001;35:186-92. | 3 |
| Vanobbergen J, Martens L, Lesaffre E, Bogaerts K, Declerck D. The value of a baseline caries risk assessment model in the primary dentition for the prediction of caries incidence in the permanent dentition. Caries Res. 2001;35:442-50. | 1 |
| Wagner Y, Heinrich-Weltzien R. Evaluation of an interdisciplinary preventive programme for early childhood caries: findings of a regional German birth cohort study. Clin Oral Investig. 2016;20:1943-52. | 1 |
| Wandera A, Bhakta S, Barker T. Caries prediction and indicators using a pediatric risk assessment teaching tool. ASDC J Dent Child. 2000;67:408-12,375. | 4 |
| Wang SS, Zhang H, Si Y, Xu T. Analysis of forecasting indexes for dental caries in 3- to 6-year-old children. Chin J Dent Res. 2016;19:153-8. | 5 |
| Wilson RF, Ashley FP. Identification of caries risk in schoolchildren: salivary buffering capacity and bacterial counts, sugar intake and caries experience as predictors of 2-year and 3-year caries increment. Br Dent J. 1989;167:99-102. | 3 |
| Yoon RK, Smaldone AM, Edelstein BL. Early childhood caries screening tools: a comparison of four approaches. J Am Dent Assoc. 2012;143:756-63. | 2 |
| Zanella-Calzada LA, Galván-Tejada CE, Chávez-Lamas NM, Gracia-Cortés MDC, Moreno-Báez A, Arceo-Olague JG, et al. A case control study of socio-economic and nutritional characteristics as determinants of dental caries in different age groups considered as public health problem: data from NHANES 2013-2014. Int J Environ Res Public Health. 2018;15:957. | 2 |
| Zhang Q, Bian Z, Fan M, van Palenstein Helderman WH. Salivary mutans streptococci counts as indicators in caries risk assessment in 6-7-year-old Chinese children. J Dent. 2007;35:177-80. | 3 |
| Zhang Q, van Palenstein Helderman WH. Caries experience variables as indicators in caries risk assessment in 6-7-year-old Chinese children. J Dent. 2006;34:676-81. | 3 |
| Zhang X, Zhang L, Zhang Y, Liao Z, Song J. Predicting trend of early childhood caries in mainland China: a combined meta-analytic and mathematical modelling approach based on epidemiological surveys. Sci Rep. 2017;7:6507 | 2 |
| Zukanović A. Caries risk assessment models in caries prediction. Acta Med Acad. 2013;42:198-208. | 6 |
