# Supplementary material for: Multivariable prediction models of caries increment: a systematic review and critical appraisal
Source: Syst Rev. 2023 Oct 30;12:202. doi: 10.1186/s13643-023-02298-y (PMC10614348; doi:10.1186/s13643-023-02298-y)
Supplement: Supplementary file 4 — Additional file 4. Detailed description of included studies as supplementary information to Table 1. [file 13643_2023_2298_MOESM4_ESM.pdf]

Additional file 4. Main characteristics of included studies of multivariable prediction models of caries increment[1]

Study<br><br>First author, year of publication [reference]	Study design	Setting<br><br>**Study dates**	Eligibility criteria<br><br>Inclusion<br><br>Exclusion	Participants at baseline<br><br>Age<br><br>Sex (% males)<br><br>Definition of caries<br><br>Caries prevalence on individual basis	Outcome to be predicted<br><br>A. Definition of outcome (caries increment)<br><br>B. Method for outcome measurement<br><br>C. Timing of outcome measurement (yrs)	Candidate predictors<br><br>Number (N)<br><br>Predictor Levels:<br>• *Societal structural*<br>• *Life-style situational*<br>• *Physiological*<br>• *Oral biological*<br>• *Tooth*<br>• *Caries experience*<br>• *Other predictors*<br><br>Predictor Levels labelled with letters which refer to additional studies describing methodology. The studies are listed in Additional file 5	Sample size<br><br>A. Number of participants (N), number of outcomes = events (E)<br><br>B. Number of events in relation to number of candidate predictors (events per variable = EPV)
**Studies of model development**							
Coronal caries							
Angulo, 1995 [13]	Cohort	Montevideo, Uruguay<br><br>1988–1990	Inclusion: children 12–13 yrs<br><br>Exclusion: NR	Age: 12–13 yrs<br><br>Sex: NR<br><br>Definition: WHO criteria, 1987 [40]<br><br>Prevalence: NR	A. DS > 1 definitive cavitation WHO criteria, 1987 [40]<br><br>B. Natural light, plane mirror, sharp probe, no radiographs<br><br>C. 1.5	N = 3<br><br>• *Oral biological*[a]<br>• *Caries experience*	A. N = 69, E = 19<br><br>B. EPV = 6.33
Demers, 1992 [14]	Cohort	Kindergarten in 15 randomly selected schools in Montréal, Canada (non-fluoridated area)<br><br>1988	Inclusion: NR<br><br>Exclusion: NR	Age: 5 yrs 8 months (± 4 months)<br><br>Sex: 52%<br><br>Definition: WHO criteria, 1979 [39]<br><br>Prevalence: NR	A. dmfs > 0 according to WHO criteria, 1979 [39]<br><br>B. Clinical with explorer by two calibrated dentists, no radiographs<br><br>C. 1	N = 9<br><br>• *Societal structural*<br>• *Life-style situational*<br>• *Physiological*<br>• *Oral biological*[b]<br>• *Caries experience*	A. N = 302, E = 143<br><br>B. EPV = 15.9

Disney, 1992 [15]	Cohort	2 areas (with cultural and ethnic differences) high caries prevalence, low fluoride in water. Aiken (SC) and Portland (ME), US<br><br>1986–1989	Inclusion: children in 1st and 5th Grade in selected schools<br><br>Exclusion: NR	Age: Aiken (Portland)<br>1st Grade: 6.6 yrs (6.9)<br>5th Grade: 10.7 yrs (10.8)<br><br>Sex:<br>Aiken (Portland)<br>1st Grade: 53% (50%)<br>5th Grade: 51% (48%)<br><br>Definition: Radike, 1972 [42]<br><br>Prevalence: NR	A. DMFS<br>Aiken: 1st Grade: ≥ 4<br>5th Grade: ≥ 5<br>Portland: 1st Grade: ≥ 2<br>5th Grade: ≥ 3<br><br>B. Clinical examination in standard manner in portable chairs and fiber optic lights, plane surface mirrors and #23 explorers, no radiographs<br><br>C. 3	Aiken N = 44<br>Portland N = 39<br><br>• *Societal structural*<br>• *Life-style situational*<br>• *Physiological*<br>• *Oral biological*[c,d]<br>• *Tooth*<br>• *Caries experience*<br>• *Other predictors*	A. Aiken: 1st Grade 1099; 5th Grade 967<br>Portland: 1st Grade 1086; 5th Grade 965<br>E: Aiken: 1st Grade 234; 5th Grade, 234<br>Portland: 1st Grade 220; 5th Grade 204<br><br>B. Aiken: 1st Grade EPV 5.3; 5th Grade EPV5.3<br>Portland: 1st Grade EPV 5.6; 5th Grade EPV 5.2
Fontana, 2011 [16]	Cohort	Rural public schools in Puerto Rico<br><br>2007	Inclusion: children with ≥1 permanent molar and ≥ 1 unrestored surface, no medical problems<br><br>Exclusion: children in need of premedication, epilepsy, non-cooperation	Age: 5–13 yrs (mean 9.7 ± 2.2)<br><br>Sex: 51%<br><br>Definition: ICDAS, 2007 [34]<br><br>Prevalence: NR	A. Two outcomes:<br>- any-progression (caries if ICDAS ≥1): presence of new lesion or new filling, and/or lesion progression from scores 1–2 (first initial signs of caries lesion) to 3 or higher (established caries), or from scores 3–4 (established caries) to 5 or higher (severe caries)<br>- progression-toward-cavitation (caries if ICDAS ≥3): presence of at least one new lesion ICDAS ≥ 3 or new filling, and/or progression of lesion from scores 1–2 to 3 or higher, or lesion progression from score 3–4 to 5 or higher<br><br>B. Clinical examination according to ICDAS [34], no radiographs<br><br>C. 1 and 2	N = 41<br><br>• *Societal structural*[e]<br>• *Life-style situational*[e]<br>• *Physiological*[e]<br>• *Caries experience*	A. N = 395,<br>E: 12 months:<br>- any-progression = 348<br>- progression-toward-cavitation = 239<br>24 months<br>- any-progression = 358<br>- progression-toward-cavitation = 268<br><br>B. 12 months<br>- any progression EPV = 8.5<br>- progression-toward-cavitation EPV = 5.8<br>24 months:<br>- any progression EPV = 8.7<br>- progression-toward-cavitation EPV = 6.5

Gao, 2010<br>[17]	Populations-based prospective study	13 randomly selected government kindergarten in Singapore, different socioeconomic profiles<br><br>2009–2010	Inclusion: children enrolled in Grade 1, including children with compromised health conditions and special needs<br><br>Exclusion: NR	Age: 3–6 yrs; Mean 4.8 (range 3.6–5.7)<br><br>Sex: 50%<br><br>Definition: WHO criteria, 1997 [41]<br><br>Prevalence: 40%	A. dmft > 0<br><br>B. Visual inspection aided by tactile inspection by one examiner, no radiographs<br><br>C. 1	Prediction models:<br>- screening N = 8<br>• *Societal structural*<br>• *Life-style situational*[j]<br>• *Physiological*[f]<br>• *Oral biological*<br>• *Caries experience*<br>- full-blown N = 12<br>• *Societal structural*<br>• *Life-style situational*[j]<br>• *Physiological*[f]<br>• *Oral biological*<br>• *Caries experience*<br><br>Risk models:<br>- screening N = 7<br>• *Societal structural*<br>• *Life-style situational*<br>• *Physiological*[f]<br>• *Oral biological*<br>• *Caries experience*<br>- full-blown N = 6<br>• *Life-style situational*<br>• *Physiological*[f]<br>• *Oral biological*<br><br>Community-screening model N = 6<br>• *Societal structural*<br>• *Life-style situational*<br>• *Physiological*[f]	A. N = 1576, E for all models = 689<br><br>B. Prediction models<br>- screening EPV = 86<br>- full-blown EPV = 57<br>Risk models<br>- screening EPV = 98<br>- full-blown EPV = 114.8<br>Community-screening model EPV = 114.6
Hänsel Petersson, 2002<br>[18]	Cohort	8 schools Halmstad, Sweden<br><br>1998	Inclusion: all schoolchildren in 22 classes of selected schools<br><br>Exclusion: children with severe disabilities, children who did not coopeate	Mean age: 10 yrs 10 months (± 12.4)<br><br>Sex: 53%<br><br>Definition: marked radiolucency with broken enamel/dentin border or with obvious progression in dentin<br><br>Prevalence: 40%	A. Change in DMFS or DMFT (> 0); dentin caries<br><br>B. Dental record review and assessment of bitewing radiographs<br><br>C. 2	N = 12<br><br>• *Societal structural*<br>• *Life-style situational*<br>• *Physiological*[f]<br>• *Oral biological*[c,g,h]<br>• *Caries experience*	A. N = 392, E = 121<br><br>B. EPV = 10

Pang, 2021 [19]	Cohort	3 urban and 3 rural schools in Foshan, China<br><br>April 2018–January 2020	Inclusion: NR<br><br>Exclusion: NR	Age: 13–14 yrs<br><br>Sex: 52%<br><br>Definition: ICDAS 2013 [35]<br><br>Prevalence: 34%	A. ICDAS codes 3–6 recorded as decayed teeth [35]<br><br>B. Clinical examination, no radiographs<br><br>C. 1.7	N = 46<br><br>• *Societal structural*<br>• *Life-style situational*[i]<br>• *Physiological*[j]<br>• *Oral biological*[k]<br>• *Tooth*<br>• *Caries experience*	A. N = 633, E = 365<br><br>B. EPV = 7.7
Sànchez-Pérez, 2009 [20]	Cohort	Public schools in southern Mexico City, Mexico<br><br>2001–2007	Inclusion: NR<br><br>Exclusion:<br>i) antibiotics within 3 weeks before sampling<br>ii) under medication	Age: 6 yrs<br><br>Sex: 48%<br><br>Definition: WHO criteria, 1997 [41]<br><br>Prevalence: 58%	A. dmfs or DMFS ≥ 1<br><br>B. Clinical examination WHO criteria, 1997 [41], no radiographs<br><br>C. 3	N = 11<br><br>• *Physiological*[l]<br>• *Oral biological*[m,n,o]<br>• *Tooth*<br>• *Caries experience*	A. N = 95, E = 56<br><br>B. EPV = 5.1
Coronal and root caries							
Powell, 1991 [21]	Cohort	Semi-independent retirement homes<br><br>NR	Inclusion: individuals >65 yrs with partial dentition<br><br>Exclusion: NR	Age: mean 80.6 yrs (66–95)<br><br>Sex: 26%<br><br>Definition:<br>- coronal caries: cavitation of enamel or "tug-back" from fissures<br>- root caries: darkened cementum/dentin and a tacky surface<br><br>Prevalence: 53%	A. ≥1 new coronal and/or root lesion<br><br>B. Clinical assessment with mirror, explorer and artificial light, no radiographs<br><br>C. 1	N = 22<br><br>• *Societal structural*<br>• *Life-style situational*<br>• *Physiological*<br>• *Oral biological*<br>• *Caries experience*	A. N = 21, E = 16<br><br>B. EPV = 0.7
Root caries							
Ritter, 2016 [22]	Retrospective cohort	Dental schools, community dental clinics, general community<br><br>2007–2008	Inclusion:<br>i) aged 21–89<br>ii) ≥12 teeth with exposed dental surfaces<br>iii) presence of at least one coronal or root surface	Age: mean 52.42 yrs (± 12.5)<br><br>Sex: 40%<br><br>Definition: RC system; a modification of ICDAS II, 2007 [34]	A. Incident RC in high-risk adults<br><br>B. Clinical examination, visual signs and tactile instrumentation<br><br>C. 3	A. 5 models (M):<br>M1 N = 8<br>• *Physiological*<br>• *Caries experience*<br><br>M2 N = 9<br>• *Life-style situational*<br>• *Physiological*<br>• *Caries experience*	A. N = 155 (only control participants included), E = 76<br><br>B.<br>M1 EPV = 9.5<br>M2 EPV = 8.4<br>M3 EPV = 5.8<br>M4 EPV = 6.9<br>M5 EPV = 6.9

			cavitated caries lesion<br><br>Exclusion:<br>i) >10 teeth with untreated lesions<br>ii) head and neck radiation or long-term antibiotics<br>iii) serious illnesses or dietary restrictions	Prevalence: 100%		M3 N = 13<br>• *Life-style situational*<br>• *Physiological*<br>• *Caries experience*<br><br>M4 N = 11<br>• *Life-style situational*<br>• *Physiological*<br>• *Caries experience*<br><br>M5 N = 11<br>• *Societal structural*<br>• *Physiological*<br>• *Caries experience*	
Sánchez-Garcia, 2011 [23]	Case-control	Family medicine unit in Mexico City, Mexico<br><br>2004–2005	Inclusion: one or more gingival recessions, free of root caries and not showing signs of treated or untreated clinical caries<br><br>Exclusion: NR	Age: mean 71.8 yrs<br><br>Sex: 32%<br><br>Definition: WHO criteria, 1997 [41]<br><br>Prevalence:<br>coronal caries 100%<br>root caries 44%	A. >1 new root surfaces with caries<br><br>B. Clinical examination; WHO criteria, 1997 [41]<br><br>C. 1	N = 31<br><br>• *Societal structural*<br>• *Life-style situational*<br>• *Physiological*[p,q,r,s]<br>• *Oral biological*[t]<br>• *Caries experience*	A. N = 531, E = 115<br><br>B. EPV = 3.7

Studies of model validation

**Study**<br><br>First author, year of publication [reference]	**Study design**	**Setting**<br><br>**Study dates**	**Eligibility criteria**<br><br>Inclusion<br><br>Exclusion	**Participants at baseline**<br><br>Age<br><br>Sex (% males)<br><br>Definition of caries<br><br>Caries prevalence on individual basis	**Outcome to be predicted**<br><br>A. Definition of outcome (caries increment)<br><br>B. Method for outcome measurement<br><br>C. Timing of outcome measurement (yrs)	**Original prediction model**<br><br>Reference to study of model development<br><br>Number of predictors (N)	**Sample size**<br><br>A. Number of participants (N), number of outcomes = events (E)<br><br>B. Number of events in relation to number of candidate predictors (events per variable = EPV)
Coronal and root caries							
Beck, 1992 [24]	Cohort	2 areas (with cultural and ethnic differences) high caries	Inclusion: children in 1st and 5th Grade in selected schools	Age: Aiken (Portland)<br>1st Grade: 6.6 (6.9)<br>5th Grade: 10.7 (10.8)<br><br>Sex: Aiken (Portland)	A. DMFS/dmfs >0 new lesion<br>- Any risk prediction model<br>- Any risk etiologic model	University of North Carolina Caries Assessment<br>Disney et al., [15]	A. N: Aiken:<br>1st Grade 1099;<br>5th Grade 967<br>Portland:<br>1st Grade 1086;

		prevalence, low fluoride in water. Aiken (SC) and Portland (ME), USA<br><br>1986–1989	Exclusion: NR	1st Grade: 53% (50%)<br>5th Grade: 51% (48%)<br><br>Definition: Radike, 1972 [42]<br><br>Prevalence: NR on individual level	B. Clinical examination in standard manner in portable chairs and fiber optic lights, plane surface mirrors, no radiographs<br><br>C. 3	Aiken N = 44<br>Portland N = 39	5th Grade 965<br>E: Aiken:<br>1st Grade 636;<br>5th Grade 642<br>Portland:<br>1st Grade 338;<br>5th Grade 443<br><br>B. Aiken:<br>1st Grade EPV = 14.4;<br>5th Grade EPV = 14.6<br>Portland:<br>1st Grade EPV = 8.7;<br>5th Grade EPV = 11.4
Birpou, 2019 [25]		Athens, Greece areas with high risk of caries	Inclusion:<br>10 preschools<br><br>Exclusion: children taking antibiotics within 2 weeks prior to enrollment or in need of premedication	Age: 2–5 yrs (mean 3.3)<br><br>Sex: NR<br><br>Definition: WHO criteria, 1997 [41]<br><br>Prevalence: 37%	A. change from "sound" to "non-cavitated lesion" or "non-cavitated " to cavitated" lesion<br><br>B. Clinical visual-tactile examination by one calibrated examiners, no radiographs<br><br>C. 1 and 2	Cariogram<br>Kavvadia et al., 2012[u]<br><br>Standard set and high set<br>Cariogram 1 and 5; N = 9 (excluding oral hygiene and saliva secretion; clinical judgement added)<br><br>Cariogram 2 and 6; N = 8 (excluding oral hygiene, saliva secretion, saliva buffer; clinical judgement added)<br><br>Cariogram 3 and 7; N = 8 (excluding ms in saliva, oral hygiene, saliva secretion; clinical judgement added)<br><br>Cariogram 4 and 7; N = 7 (excluding ms in saliva, oral hygiene, saliva secretion, saliva buffer; clinical judgement added)	A. N = 147 (1 yrs)<br>N = 140 (2 yrs)<br>E = 77 (1 yrs)<br>E = 74 (2 yrs)<br><br>B. 1 yrs<br>Standard set and high set<br>Cariogram 1 and 5<br>EPV = 8.5<br>Cariogram 2 and 6<br>EPV = 9.6<br>Cariogram 3 and 7<br>EPV = 9.6<br>Cariogram 4 and 8<br>EPV = 10<br><br>2 yrs<br>Standard set and high set<br>Cariogram 1 and 5<br>EPV = 8.2<br>Cariogram 2 and 6<br>EPV = 9.25<br>Cariogram 3 and 7<br>EPV = 9.25<br>Cariogram 4 and 8<br>EPV = 10.1
Campus, 2012 [26]	Cluster sample calculated on	Sardinia, Italy in area with low natural	Inclusion: schoolchildren 7–	Age: 7–9 yrs<br><br>Sex: 49%	A. Caries increment – DFS increment of decayed and	Cariogram<br>Bratthall & Hänsel Petersson, 2005[v]	A. N = 861, E = 469<br><br>B. EPV = 67

	caries prevalence in studied area	fluoride content, children reporting use of fluoride toothpaste at least twice daily<br><br>January to June 2007-2009	9 yrs in different school-classes<br><br>Exclusion: NR	Definition: Pitts & Longbottom, 1995 [43]<br><br>Prevalence: 29%	filled permanent tooth surfaces.<br><br>B. Clinical visual-tactile examination by 2 calibrated examiners, no radiographs<br><br>C. 2	N = 7 (excluding saliva buffer, saliva secretion)	
Christian, 2020 [27]	Cohort	Victoria, Australia<br><br>NR	Inclusion: participants in an oral health birth cohort<br><br>Exclusion: NR	Age: 18 months<br><br>Sex: NR<br><br>Definition: ICDAS-II 2007 [34]<br><br>Prevalence: 0%	A. ICDAS-II score 1–6 [34]<br><br>B. Clinical examination, no probe, no radiographs<br><br>C. 1.5 and 2.5	Age-modified model of CAMBRA Featherstone et al., 2003[w]<br><br>N = 13	A. N = 214, E: 1.5 yrs = 39 E: 2.5 yrs = 75<br><br>B. 1.5 yrs EPV = 3.0 2.5 yrs EPV = 5.8
Dolic, 2020 [28]	Cohort	Banja Luka, Bosnia-Hercegovina<br><br>2007–2011	Inclusion: women in the last trimester of a normal single-fetus pregnancy<br><br>Exclusion:<br>i) no earlier high-risk pregnancies<br>ii) no previous medical conditions, pregnancy complications, or pregnancy-related issues that required hospitalization<br>iii) no chronic disease<br>iv) no antibiotics or other drugs	Age: mean 27.4 ± 7.2 yrs<br><br>Sex: 0%<br><br>Definition: WHO criteria 1987 [40]<br><br>Prevalence: NR	A. DT > 1 definitive cavitation WHO criteria (1987) [40]<br><br>B. Visual and tactile examination, no radiographs<br><br>C. 4	Cariogram Bratthall & Hänsel Petersson, 2005[v]<br><br>N = 9	A. N = 80, E = 54<br><br>B. EPV = 6

			during pregnancy				
Gao, 2013 [29]	Cohort	Hong Kong, areas with different socioeconomic profiles<br><br>Study dates NR	Inclusion: children from 4 kindergartens<br><br>Exclusion: un-cooperation and severe medical conditions	Age: 3 yrs<br><br>Sex: 52%<br><br>Definition: WHO criteria 1997 [41]<br><br>Prevalence: 34.7%	A. dmft > 0 WHO criteria for caviation [41]<br>CAT and CAMBRA (low, moderate and high risk)<br>CARIOGRAM and NUS-CRA on a continuous scale<br>Cariogram 5 risk groups according to chance of avoiding caries:<br>81–100% (very low–low risk);<br>61–80% (low);<br>41–60% (moderate);<br>21–40% (high);<br>0–20% (very high–high risk)<br><br>B. Clinical visual-tactile examination by 1 examiner, no radiographs<br><br>C. 1	NUS-CRA:<br>Gao et al., 2010 [17]<br>a) comprehensive N = 11<br>b) screening N = 9 (excluding ms in saliva, lbc in saliva)<br><br>Cariogram<br>Bratthall & Hänsel Petersson, 2005[v]<br>c) comprehensive N = 9<br>d) screening N = 5 (excluding ms in saliva, lbc in saliva, saliva buffer, saliva secretion)<br><br>CAT<br>Reference Manual 2006[x]<br>e) comprehensive N = 12<br>f) comprehensive N = 11 (excluding family economic status)<br>g) screening N = 10 (excluding ms in saliva, saliva secretion)<br>h) screening N = 9 (excluding ms in saliva, saliva secretion, family economic status)<br><br>CAMBRA<br>Ramos-Gomez et al., 2007[y]<br>i) comprehensive N = 14<br>j) screening N = 11 (excluding ms in saliva, lbc in saliva, saliva secretion)	A. N = 485, E total = 178<br><br>B.<br>a) NUS-CRA<br>EPV = 16.2<br>b) NUS-CRA<br>EPV = 19.8<br>c) Cariogram<br>EPV = 19.8<br>d) Cariogram<br>EPV = 35.6<br>e) CAT<br>EPV = 16.2<br>f) CAT<br>EPV = 17.8<br>g) CAT<br>EPV = 17.8<br>h) CAT<br>EPV = 19.8<br>i) CAMBRA<br>EPV = 12.7<br>j) CAMBRA<br>EPV = 16.2
Hänsel Petersson, 2015 [30]	Cohort	8 selected public dental health clinics,	Inclusion: adolescents registered in selected clinics	Age: 19 yrs<br><br>Sex: 54%	A. DFS ≥ 1 carious lesion (from sound to decayed or filled) in 5 groups according to chance of avoiding caries:	Cariogram<br>Hänsel Peterson et al., [18]	A. N = 982<br>N in each category:<br>81–100% 251;<br>61–80% = 335;

		Skåne,<br>Sweden<br><br>2006–2007	Exclusion: studied/lived abroad, attended a specialist clinic, attended private care, did not consent	Definition: WHO criteria 1987 [40]<br><br>Prevalence: 77%	81–100% (very low);<br>61–80% (low);<br>41–60% (moderate);<br>21–40% (high);<br>0–-20% (very high)<br><br>B. Clinical visual-tactile examination,<br>bitewing radiographs<br><br>C. 3	N = 9	41–60% = 248;<br>21–40% = 74;<br>0–20% = 74<br>E:<br>81–100% = 37;<br>61–80% =96<br>41–60% = 123<br>21–40% = 47;<br>0–20% = 41<br><br>B.<br>81–100% EPV = 4.1<br>61–80% EPV = 10.66<br>41–60% EPV = 13.66<br>21–40% EPV = 5.2<br>0–20% EPV = 4.55
Hänsel Petersson, 2010<br>[31]	Cohort	8 selected schools in a city Sweden<br><br>1998–2000	Inclusion: school-children<br><br>Exclusion: severe disabilities and children who did not cooperate in an oral inspection	Age: 10 yrs 10 months (± 12.4 months)<br><br>Sex: 53%<br><br>Definition: radiolucency with broken enamel-dentin border or obvious progression in dentin<br><br>Prevalence: 40%	A. DMFS > 0<br>from sound to decayed or filled<br><br>B. Data extracted from dental records and bitewing radiographs<br>Clinical visual-tactile examination, bitewing radiographs<br><br>C. 2	Cariogram<br>Hänsel Peterson et al., [18]<br><br>a) Full Cariogram N = 9<br>b) Reduced Cariogram N = 8<br>(excluding either ms in saliva, saliva buffer or saliva secretion)<br>c) Reduced Cariogram N = 6<br>(excluding ms in saliva, saliva buffer, saliva secretion)	A. N = 392, E = 122<br><br>B.<br>a) Cariogram<br>EPV = 13.5<br>b) Reduced Cariogram<br>EPV = 15<br>c) Reduced Cariogram<br>EPV = 20.3
Holgerson, 2009<br>[32]	Cohort	Public Dental Health clinic in a small city, Sweden<br><br>2002–2007	Inclusion: children born 2000-01<br><br>Exclusion: severe disabilities, non-cooperation with oral inspection	Age: 2 yrs<br><br>Sex: NR<br><br>Definition: WHO criteria [41] modified for primary dentition<br><br>Prevalence: 3%	A. dmfs/DMFS > 0 including non-cavitated lesions<br><br>B. Clinical and partly radiographic examination (84% of the sample)<br><br>C. 5	Cariogram:<br>Bratthall, 1996[z]<br><br>N = 7 (excluding saliva buffer, saliva secretion, diet content, clinical judgement added after result of Cariogram was obtained)	A. N = 55 (only control group included), E = 20<br><br>B. EPV = 2.9
Pang, 2020<br>[19]	Cohort	3 urban and 3 rural schools, Foshan, China	Inclusion: NR<br><br>Exclusion: NR	Age: 13–14 yrs<br><br>Sex: 52%<br><br>Definition:	A. Codes 3–6 in ICDAS system recorded as decayed teeth<br><br>B. Clinical examination,	Model after Pang et al., [19]<br><br>N = 46	A. N = 320, E = 202<br><br>B. EPV = 4.4

		April 2018–<br>January 2020		ICDAS 2013 [35]<br><br>Prevalence: 40%	no radiographs<br><br>C. 1.7		
**Root caries**							
Hayes, 2018 [33]	Cohort	- Cork, community, Ireland<br>- Cork dental school<br><br>2012–2015	Inclusion:<br>i) minimum one natural tooth<br>ii) living independently<br>iii) cognitive ability to understand consent procedure<br><br>Exclusion:<br>i) patients living in nursing home<br>ii) antibiotics previous month	Age: ≥ 65 yrs<br><br>Sex: NR<br><br>Definition: Modification of ICDAS 2007 [34]<br><br>Prevalence: NR	A. Number of root surfaces which developed a new cavitated lesion<br><br>B. Clinical examination ICDAS [34]<br><br>C. 2	Cariogram<br>Bratthall Cariogram manual, 2004[aa]<br><br>a) Cariogram N = 9<br>b) Reduced Cariogram N = 8 (excluding ms in saliva)<br>c) Reduced Cariogram N = 8 (excluding saliva buffer)<br>d) Reduced Cariogram N = 8 (excluding saliva secretion)<br>e) Reduced Cariogram N = 6 (excluding ms, saliva buffer, saliva secretion)	A. N = 280, E = 70<br><br>B. a) Cariogram EPV = 7.8<br>b, c, d) Reduced Cariogram EPV = 8.8<br>e) Reduced Cariogram EPV = 11.7

Notes: [1]Data extraction used a protocol based on CHARMS [4]

Abbreviations: *ms*, mutans streptococci; *lbc*, lactobacilli; *DMFT,* decayed missing filled teeth (permanent); *DMFS,* decayed missing filled surfaces (permanent); *DS*, decayed surfaces (permanent); *dmft*, decayed missing filled teeth (primary); *dmfs,* decayed missing filled surfaces (primary); *saliva buffer,* saliva buffering capacity; *saliva secretion,* saliva secretion flow rate; *NR*, not reported
