# Supplementary material for: Multivariable prediction models of caries increment: a systematic review and critical appraisal
Source: Syst Rev. 2023 Oct 30;12:202. doi: 10.1186/s13643-023-02298-y (PMC10614348; doi:10.1186/s13643-023-02298-y)
Supplement: Supplementary file 5 — Additional file 5. Reference list of publications describing methodology for predictors presented in Additional file 4. [file 13643_2023_2298_MOESM5_ESM.pdf]

Additional file 5. Reference list of additional studies describing methodology of predictors presented in Additional file 4.

a. Westergren G, Krasse B. Evaluation of micromethod for determination of *Streptococcus mutans* and *Lactobacillus* infection. J Clin Microbial. 1978;7:82-3.
b. Green JC, Vermillion JR. The simplified oral hygiene index. J Am Dent Assoc. 1964;68:7-13.
c. Larmas M. A new dip-slide method for the counting of salivary lactobacilli. Proc Finn Dent Soc. 1975;71:31-5.
d. Jordan HV, Laraway R, Snirch R, Marmel M. A simplified diagnostic system for cultural detection and enumeration of *Streptococcus mutans.* J Dent Res. 1987;66:57-61.
e. Fontana M, Jackson R, Eckert G, Swigonski N, Chin J, Ferreira Zandona A, et al. Longitudinal assessment of caries risk factors in toddlers. J Dent Res. 2010;90:209-14.
f. Silness J, Löe H. Periodontal disease in pregnancy. II. Correlation between oral hygiene and periodontal condition. Acta Odontol Scand. 1964; 22:121-35.
g. Jensen B, Bratthall D. A new method for estimation of mutans streptococci in human saliva. J Dent Res. 1989;68:468-71.
h. Ericson D, Bratthall D. Simplified method to estimate salivary buffer capacity. Scand J Dent Res. 1989;97:405-7.
i. Wang K, Pang L, Fan C, Cui T, Yu L, Lin H. Enamel and dentin caries risk actors of adolescents in the context of the International Caries Detection and Assessment System (ICDAS): a longitudinal study. Front Pediatr. 2020;8:419.
j. Löe, H. The gingival index, the plaque index, and the retention index systems. J Periodontol. 1967;38:610-6.
k. Wang K, Pang L, Tao Y, Li X, Zhang J, Cui T, et al. Association of genetic and environmental factors with dental caries among adolescents in south China: a cross-sectional study. Eur J Paediatr Dent. 2020;21:129-36.
l. Graves RC, Abernathy JR, Disney JA, Stamm JW, Bohannan HM. University of North Carolina caries risk assessment study. III. Multiple factors in caries prevalence. J Public Health Dent. 1991;51:134-43.
m. Rogosa M, Mitchell JA, Wiseman R. A selective medium for the isolation and enumeration of oral lactobacilli. J Dent Res. 1951; 30: 682-9.
n. Snyder ML. Laboratory methods in the clinical evaluation of caries activity. J Am Dent Assoc. 1951; 42: 400-13.
o. Juárez Tomás MS, Bru de Labanda E, de Ruiz Holgado AP, Nader-Macías ME. Estimation of vaginal probiotic lactobacilli growth parameters with the application of the Gompertz model. Can J Microbiol. 2002;48: 82-92.
p. Katz S, Ford AB, Moskowitz RW, Jackson BA, Jaffe MW. Studies of illness in the aged. The index of ADL: a standardized measure of biological and psychosocial function. JAMA. 1963;185:914-9.
q. Lawton MP, Brody EM. Assessment of older people: self-maintaining and instrumental activities of daily living. Gerontologist. 1969;9:179-86.
r. Reyes-Beaman S, Beaman PE, Garcıa-Pena C, Villa MA, Heres J, Cordova A et al. Validation of a modified version of the Minimental State Examination (MMSE) in Spanish. Aging Neuropsychol Cogn. 2004;11:1-11.

s. Reyes S. Population ageing in the Mexican Institute of Social Security: health policy and economic implications. Ed. IMSS-Fundacion Mexicana para la Salud. Mexico, 2001; available at: http:// www.funsalud.org.mx/quehacer/publicaciones/popageing/popageing.htm [last accessed 10 August 2009].
t. Sanchez-Garcıa S, Gutierrez-Venegas G, Juarez Cedillo T, Reyes-Morales H, Solorzano-Santos F, Garcıa-Pena C. A simplified caries risk test in stimulated saliva from elderly patients. Gerodontology. 2008;25:26-33.
u. Kavvadia K, Agouropoulos A, Gizani S, Papagiannouli L, Twetman S. Caries risk profiles in 2- to 6- year- old Greek children using the Cariogram. Eur J Dent. 2012;6:415-21.
v. Bratthall D, Hänsel Petersson G. Cariogram – a multifactorial risk assessment model for a multifactorial disease. Community Dent Oral Epidemiol. 2005;33:256-64.
w. Featherstone JD, Adair SM, Anderson MH, Berkowitz RJ, Bird WF, Crall JJ, et al. Caries management by risk assessment: consensus statement, April 2002. J Calif Dent Assoc. 2003;31: 257-69.
x. American Academy of Pediatric Dentistry. Policy on use of a caries-risk assessment tool (CAT) for infants, children, and adolescents. Reference Manual V 30/No7 08/09 (2006).
y. Ramos-Gomez FJ, Crall J, Gansky SA, Slayton RL, Featherstone JD. Caries risk assessment appropriate for the age 1 visit (infants and toddlers). J Calif Dent Assoc. 2007;35:687-702.
z. Bratthall D. Dental caries: intervened interrupted interpreted. Concluding remarks and Cariography. Eur J Oral Sci. 1996;104:486-91.
aa. Bratthall D, Hänsel-Petersson G, Stjernswärd J, Cariogram manual, Cariogram, Internet Version 2, (2004). [cited 2022 Feb 02]. Available from: https://mau.se/om-oss/fakulteter-och-institutioner/odontologiska-fakulteten/sektioner-content-grid/sektion-3/#accordion-64634
