# Supplementary material for: Multivariable prediction models of caries increment: a systematic review and critical appraisal
Source: Syst Rev. 2023 Oct 30;12:202. doi: 10.1186/s13643-023-02298-y (PMC10614348; doi:10.1186/s13643-023-02298-y)
Supplement: Supplementary file 7 — Additional file 7. Distribution of predictors by Level and category in multivariable developmental models of coronal caries increment. Predictors included in final models highlighted in red. [file 13643_2023_2298_MOESM7_ESM.pdf]

Additional file 7. Distribution of predictors by Level and category in multivariable developmental models of coronal caries increment. Predictors included in final models highlighted in red.

SOCIETAL STRUCTURAL LEVEL	***Predictor***
**Demography**	**[7]** residence
**Parents' education**	**[2–M1, M2]** parents' education; **[3]** education household head **[4–M1-M11]** caregiver´s education (high school vs college degree), caregiver´s education (post high school vs college degree), caregiver has difficulty in understanding information from health care professional, caregiver has difficulty in communicating with health care professional, **[5–M1,M2]** father; **[7]** education of caregiver
**Family income**	**[7]** family income
**Parents' oral health**	**[4–M1-M11]** caregiver has caries
**Parents' view of child's health**	**[4–M1-M10]** caregiver does not consider child´s oral health to be very good; **[5–M5]** tooth worm, bedtime milk, child´s dental status according to parents
**Family structure**	**[2–M2]** family structure; **[3]** number of other children living in the household. **[7]** one-child family
**Referral system in oral health care**	**[3]** referral caries score; **[4–M3, M9, M10, M11]** caregiver received referral for child, **[4–M1-M11]** caregiver followed up referral; caregiver took referral to the dentist
**Fluoridated community**	**[5–M3]** never lived in non-fluoridated community
**School**	**[6]** school
**Dental Clinic**	**[6]** dental clinic
LIFE-STYLE SITUATIONAL LEVEL	***Predictor***
**Fluoride supplements**	**[2–M2, 6]** fluoride supplements; **[3]** F-rinse; **[4–M1-M11]** uses more toothpaste; no use of additional fluoride at home regular; professional fluoride; occasional professional fluoride; **[5–M2, M5]** using fluoride
**Food intake frequency**	**[3]** snacks between-meals; **[4–M3]** soda between meals; **[5–M1, M3]** sweets between meals<br>**[5–M2, M3]** bedtime feeding; bedtime sweets; **[6]** diet frequency<br>**[4–M3]** child soda between meals; **[4–M1-11]** child drinks water; child drinks tap water vs. well water; child drinks tap water vs. bottled water; no drinks of juice between meals; use of snacks between meals; use of sugar-containing gum; sweet drinks between meals >2 x/day vs. never; sweet drinks between meals >2 x/day vs. 1 x/day sweet drinks between meals >2 x/day vs. 2 x/day; use of snacks between meals >2 x/day vs. never; use of snacks between meals >2 x/day vs. 1 x/day; use of snacks between meals >2 x/day vs. 2 x/day; use of sugar-containing gum > 2 x/day vs. never; use of sugar-containing gum > 2 x/day vs. 1 x/day; use of sugar-containing gum > 2 x/day vs. 2 x/day
**Oral hygiene routines**	**[3]** tooth brushing; **[4–M1-M11]** brushes less twice a day; **[7]** frequency of tooth brushing
**Dental attendance**	**[3]** household head number of dental visit in past year, number of times child visited dentist in past year, reason for dental visit; **[4–M1, M2, M3, M6, M7, M8]** child has been to the dentist in past 3 months vs 6 months; **[5–M2]** annual dental checks
**Infant feeding**	**[5–M1, M2, M3, M4]** months of breastfeeding
**Diet content (lbc in saliva)**	**[6]** diet content (lbc in saliva)

PHYSIOLOGICAL LEVEL	*Predictor*
Age	[3] age; [4–M1-M11] age; [5–M1-M5] age
Ethnicity	[3] race, [5–M1, M5] race
General health	[3] ear infection history, strep throat history, antibiotics in past 60 days; [4–M1-M11] child not covered under health care reforms; [5–M1, M2] no health problems; [6] related disease
Sex	[2–M1, M2] sex; [3, 8] gender; [4–M1-M11] female; [6, 7] sex
ORAL BIOLOGICAL LEVEL	*Predictor*
Mutans streptococci (ms) in saliva	[1] ms in saliva; [2–M1] ms in saliva; [3] ms in saliva; [5–M2, M4] ms in saliva; [6] ms in saliva
Lactobacilli (lbc) in saliva	[1] lbc in saliva; [2–M1] lbc in saliva; [3] lbc in saliva; [5–M2, M4] lbc in saliva
ms and lbc in dental plaque	[8] ms and lbc in dental plaque
Visible dental plaque	[2–M1] debris index; [3] plaque score; [5–M1, M2, M3, M4] plaque index; [6] plaque amount; [7] plaque index
Acid production salivary bacteria	[8] acid production salivary bacteria
Saliva secretion	[6] saliva secretion; [8] salivary flow rate
Saliva buffer	[2–M1, M2] saliva buffer; [5–M2, M4] pH; [6] saliva buffer
Cariostate score	[7] cariostate score
TOOTH LEVEL	*Predictor*
Number of teeth	[8] number of teeth
Tooth morphology	[3] tooth morphology; [8] fissure morphology
Fluorosis	[3] fluorosis
Sound permanent tooth surfaces	[3] sound permanent tooth surfaces
Sealants	[3] sealants
Genetic markers for enamel and Water channel protein	[7] rs3790506, rs1996315, rs10779570, rs11003125, rs1126478, rs11362, rs12640848, rs13115627, rs134143, rs1612069, rs17640579, rs1784418, rs1800450, rs1800972, rs2097470, rs2274327, rs35874116, rs3796703, rs457741, rs713598, rs923911, rs946252, rs9701796
CARIES EXPERIENCE	*Predictor*
Cavitation	[6] DMFT; [7] ICDAS 3-6; [5–M1, M2] dmft; [2–M1] dmfs; [3] dmfs; [4–M2, M7, M10] ICDAS≥3 at end; [4–M3, M5, M8, M11] ICDAS≥3 at start; [8] dmfs; [3] DMFS; [4–M2, M10] ICDAS≥3 at end; [4–M3, M5, M8, M11] ICDAS≥3 at start; [8] DMFS; [1] DS; [4–M9, M10, M11] current caries; [4–M6, M7, M8, M9, M10, M11] tooth restored; [4–M1, M2, M3] tooth extracted + tooth restored; [4–M4, M5] tooth extracted
White spot lesion	[3] White spot lesion
OTHER TYPES OF PREDICTORS	*Predictor*
Examiner´s judgement	[3] examiner’s score; predicted caries score
Combinations	[3] fluorosis x white spot lesion
Excluded predictors	[3] other referral score; use of fluoride drops; fluoride tablets or vitamins, visual dental plaque X fissure morphology, visual dental plaque X ms in saliva, visual dental plaque X lbc in saliva, ms in saliva X white spot lesion, lbc in saliva X

	white spot lesion, ms in saliva X fissure morphology, lbc in saliva X fissure morphology, race X education, race X age, race X other children and race X sex [4–M1-M11] caregiver believes child does not have a 'very good' medical health; caregiver supervises child´s tooth brushing regularly; caregiver supervises child´s tooth brushing occasionally; child has been to the dentist in past 3-6 months vs >6 months; [4–M1, M2, M4-M8] caregiver received referral for child; [4–M4, M5, M9-M11] child has been to the dentist in past 3 months vs 6 months; [4–M1, M2, M4-M11] child soda between meals
REFERENCES<br>1. Angulo, 1995 [13]<br>2. Demers, 1992 [14]<br>3. Disney, 1992 [15]<br>4. Fontana, 2011 [16]<br>5. Gao, 2010 [17]<br>6. Hänsel Petersson, 2002 [18]<br>7. Pang, 2021 [19]<br>8. Sánchez-Pérez, 2009 [20]	 One model / [12-13 years]<br>Two models (M1, M2) / [5-8 years]<br>One model [6-10 years] / [6-10 years]<br>Eleven models (M1-M11) / [5-13 years]<br>Five models (M1-M5) / [12-13 years] / [3-6 years]<br>One model / [10 years]<br>One model / [13-14 years]<br>One model / [6 years]
